# A Systematic Review of Hypnosis for Clinical Pain Relief

**DOI:** 10.1002/ejp.70369

**Published:** 2026-09-02

**Authors:** Titus H. Yim, Stuart W. G. Derbyshire

**Affiliations:** ^1^ Department of Psychology National University of Singapore Singapore

## Abstract

**Background:**

Previous reviews report moderate reductions in clinical pain with hypnotic suggestion but provide only partial views by comparing hypnosis with inactive controls alone or including few studies or mixed control groups. This review comprehensively evaluated the efficacy of hypnosis in reducing clinical pain intensity compared with non‐active and active controls.

**Methods:**

Systematic review following Preferred Reporting Items for Systematic Reviews and Meta‐Analyses (PRISMA) review guidelines with comprehensive literature search and meta‐analysis. A total of 106 studies met inclusion criteria; 104 effect sizes from 84 studies were pooled using random‐effects models, and 22 studies were narratively synthesised. Five databases were searched from inception to October 2025 for randomised controlled trials evaluating hypnosis versus non‐active or active non‐pharmacological controls for clinical pain intensity. Two independent reviewers screened articles and extracted data using standardised tools.

**Results:**

Meta‐analysis of 57 studies (4572 participants) showed reduced post‐intervention pain following hypnosis versus non‐active controls (SMD −0.33). Pre–post change scores yielded a comparable but non‐significant effect (SMD −0.31). Subgroup analyses showed no differences by pain type, control type or delivery mode. Hypnosis did not outperform active controls, including relaxation (14 studies, SMD −0.13), pain education (7 studies, SMD −0.17 to −0.19) or cognitive‐behavioural therapy (5 studies, SMD −0.32).

**Conclusions:**

Hypnosis yields modest reductions in clinical pain comparable to other psychological interventions.

**Significance Statement:**

This review provides a comprehensive synthesis of randomised trials evaluating hypnosis for clinical pain intensity, distinguishing non‐active from active comparators and examining delivery mode, pain type and risk of bias. Hypnosis produced only small reductions in pain and did not clearly outperform relaxation, pain education or cognitive‐behavioural therapy. These findings refine expectations for hypnoanalgesia, suggesting it may provide low‐risk supportive care for selected patients, but should not be presented as a reliably superior pain‐reduction treatment option.

**Trial Registration:**

Prospero (CRD42024608722)

## 1 Introduction

Chronic pain imposes a substantial global burden, with prevalence estimates ranging from 20% to 40% that generate major contributions to disability and economic costs approaching $600 billion annually (GBD 2016 Disease and Injury Incidence and Prevalence Collaborators 2017; Cohen et al. 2021; Jackson et al. 2016; U.S. Burden of Disease Collaborators 2013). Despite extensive pharmacological treatment, many patients achieve inadequate relief and concerns about opioid‐related harms have increased interest in safer psychological approaches (Sullivan and Ballantyne 2023).

Non‐pharmacological interventions such as cognitive behavioural therapy (CBT) and acceptance‐based therapies typically yield modest reductions in pain intensity, averaging approximately one point on a 0–10 scale (Williams et al. 2020; Venkiteswaran and Tandon 2021; Jenkins et al. 2025). These effects are often borderline clinically meaningful and do not consistently exceed placebo controls (Ferreira et al. 2012; Strijkers et al. 2021), although some patients experience broader benefits in emotional or functional outcomes.

Hypnosis has similarly been reported to reduce acute, procedural, and chronic pain by roughly one point on a 0–10 scale (Adachi et al. 2014; Langlois et al. 2022; Rosendahl et al. 2024; Yerzhan et al. 2025), with considerable variability across studies. As an additional intervention, hypnosis has shown modest enhancement of pain education or pharmacological treatments, though effects diminish when controlling for time and attention (Jones et al. 2024). Prior meta‐analyses, however, have often included small samples, pooled heterogeneous control conditions or focused exclusively on placebo comparisons or additional effects, limiting clinical interpretability (Ramondo et al. 2021; Langlois et al. 2022; Jones et al. 2024; Rosendahl et al. 2024; Yerzhan et al. 2025).

The primary aim of the present meta‐analysis was to evaluate the efficacy of hypnosis in reducing clinical pain intensity compared with non‐active controls (e.g., waitlist, attention or treatment as usual) and relaxation‐based interventions. Second, we compared hypnosis with other active non‐pharmacological treatments (CBT and pain education). Finally, we examined whether delivery modality (recorded, therapist‐delivered, self‐hypnosis or mixed) and pain type (acute vs. chronic) moderated treatment effects.

## 2 Methods

The review protocol was prospectively registered on PROSPERO (ID: CRD42024608722) and conducted in accordance with PRISMA guidelines (Moher et al. 2009) (see Data S1 for the PRISMA checklist).

### 2.1 Search Strategy

Two consecutive searches (initial and updated) were conducted across PsycINFO, PubMed, Scopus, Web of Science and the Cochrane Library Register of Controlled Trials from database inception to 20 October 2025. Search terms combined variants of hypnosis and non‐pharmacological intervention with terms related to pain reduction (full search strategy in Data S5). Searches were applied to titles, abstracts and keywords (Scopus: title only).

#### 2.1.1 Eligibility Criteria

The full inclusion and exclusion criteria are detailed in Table 1.

**TABLE 1:** Inclusionary and exclusionary criteria.

	Inclusion criteria	Exclusion criteria
Participants	Adults (mean age ≥ 18 years or older).	Children (mean age < 18 years of age) or patients deliberately selected as high (or low) hypnosis responders
Intervention	Studies directly assessing hypnosis to deliver pain relief with or without specific suggestions for pain relief and delivered in any format (e.g., in‐person or via a pre‐recorded audio file, individually or in a group setting, alone or as a supplement to other forms of therapy) The study will be considered a hypnosis study if the authors specifically refer to the procedure as involving hypnosis, or the patients specifically consent to being hypnotised, or the study clearly includes an induction procedure intended to induce a hypnotic ‘trance’ state.	Interventions carried out in the study do not include or do not adequately represent core hypnosis or suggestion elements.
Type of pain	Chronic or acute clinical pain.	Experimentally‐induced pain.
Type of publication	Peer‐reviewed journal article.	Non‐journal articles (e.g., book, conference proceedings and non‐peer‐reviewed articles)
Study design	Randomised controlled studies with an experimental (hypnosis) group versus a control group (i.e., passive control or active control)	Studies of a descriptive/qualitative nature that do not report empirical results, single‐session laboratory studies, case studies, studies with non‐experimental designs or any forms of reviews (i.e., systematic reviews, scoping reviews, narrative reviews and meta‐analyses)
Outcome measures	Outcome measures for the intervention are reported. Studies that utilise a Visual Analogue Scale (VAS) to measure pain. Studies can either present pre‐intervention‐post‐intervention measures or post‐intervention measures only	Questionnaires with aggregated pain scores or those comprising multiple items about pain (e.g., Brief Pain Inventory, McGill Pain Questionnaire) that do not report separately on pain intensity

### 2.2 Target Population

Eligible studies included adults (≥ 18 years) experiencing acute or chronic clinical pain. Studies involving experimentally induced pain, participants under 18 years, or samples selected for high or low hypnotic responsiveness were excluded.

### 2.3 Study Design and Publication Type

We included peer‐reviewed randomised controlled trials (RCTs) comparing hypnosis with non‐active/minimal controls (e.g., waitlist, attention and treatment as usual) or active comparators. Non‐randomised studies, studies without control groups, qualitative or descriptive designs, single‐session laboratory studies, case reports and review articles were excluded. Studies in all languages were eligible and translated where required.

### 2.4 Intervention

Included studies directly evaluated hypnosis for reducing pain intensity, with or without specific analgesic suggestions, delivered in any format (e.g., therapist‐delivered, audio‐recorded, self‐hypnosis, individual or group). Studies in which hypnosis was combined with another intervention, combined therapies (e.g., hypnosis with CBT), were included only when an appropriate control was included (e.g., hypnosis alone or CBT alone) to allow for the additional contribution of hypnosis or the other intervention to be evaluated.

### 2.5 Outcome Measures

Studies were required to report pain intensity using a Visual Analogue Scale (VAS) or Numerical Rating Scale (NRS). Multi‐item pain questionnaires not reporting pain intensity separately were excluded. Studies had to provide means and standard deviations for post‐intervention scores and/or pre–post change scores; studies lacking sufficient variance data were excluded.

### 2.6 Study Selection and Data Extraction

Screening was conducted by the first author (TY) and verified by the senior author (SD). Title/abstract and full‐text screening were performed independently by both authors, with disagreements resolved by consensus. Inter‐rater reliability was assessed using Cohen's kappa (McHugh 2012).

Data were extracted using a standardised spreadsheet and verified by both authors. Extracted variables included study characteristics (author, year and country), sample size and demographics, pain type, intervention characteristics, comparator type, number of sessions, pain measures and pain intensity outcomes (means and standard deviations or effect sizes). Hypnosis modality was coded a priori (see Data S5). Information required for risk of bias assessment was also extracted. Unreported data were requested from study authors.

### 2.7 Risk of Bias Assessment

Risk of bias (RoB) was independently assessed by both authors, with discrepancies resolved by discussion. Inter‐rater agreement was evaluated using Cohen's kappa (McHugh 2012).

A 21‐item RoB tool was applied, based on the Revised Cochrane RoB tool for randomised trials (Sterne et al. 2019) and the validity scale developed by Thompson et al. (2019). The tool assessed methodological rigour, selection processes and reporting bias, including factors particularly relevant to hypnosis research (e.g., hypnotisability measurement, medication use, treatment expectations and intervention fidelity). Items were rated as ‘yes’, ‘no’ or ‘uncertain/unknown’. Overall RoB was classified as low (≥ 15), moderate (11–14) or high (≤ 10). Full item ratings are provided in Data S2.

Overall certainty of evidence was evaluated using the GRADE framework, which provides a structured approach for judging how much confidence should be placed in an estimated treatment effect. With randomised trials initially rated as high‐certainty evidence, this certainty may then be downgraded when there are important concerns about risk of bias, inconsistency, indirectness, imprecision and publication bias domains (Prasad 2024).

### 2.8 Data Preparation

Non‐standard VAS scales (e.g., 0–240; VanDyck et al. 1991) were rescaled to a 10‐point metric prior to analysis. No distinction was made between 10‐point and 11‐point scales. The zero in 11‐point scales indicates ‘no pain,’ which is rarely reported, and the one in both scales refers to a comparable minimal level of pain.

### 2.9 Statistical Analysis

Given anticipated heterogeneity, random‐effects meta‐analyses were conducted. Between‐study variance (τ^2^) was estimated using restricted maximum likelihood (Viechtbauer 2005). Heterogeneity was assessed using Cochran's Q and *I* ^2^ statistics. Effect sizes were calculated as Hedges' g with 95% confidence intervals (Hedges and Olkin 1984), applying the Knapp–Hartung adjustment to account for small sample bias (Knapp and Hartung 2003; IntHout et al. 2014; Langan et al. 2019). Analyses were conducted in R using the *metafor* package (Viechtbauer 2010; R Core Team 2017).

Separate meta‐analyses were conducted for studies reporting post‐intervention scores and those reporting pre–post change scores. Subgroup analyses examined risk of bias, pain type (acute vs. chronic) and hypnosis modality where sufficient studies were available. Studies not suitable for pooling were narratively synthesised (see Data S5).

### 2.10 Publication Bias and Sensitivity Analyses

Publication bias and small study effects were assessed using funnel plots for analyses including ≥ 10 studies (Sterne et al. 2011), and Egger's regression test for analyses including ≥ 30 studies (Lau et al. 2006), respectively. Leave‐one‐out analyses were conducted for studies with large or anomalous effect sizes and moderate‐to‐high RoB to assess robustness.

## 3 Results

**FIGURE 1:**
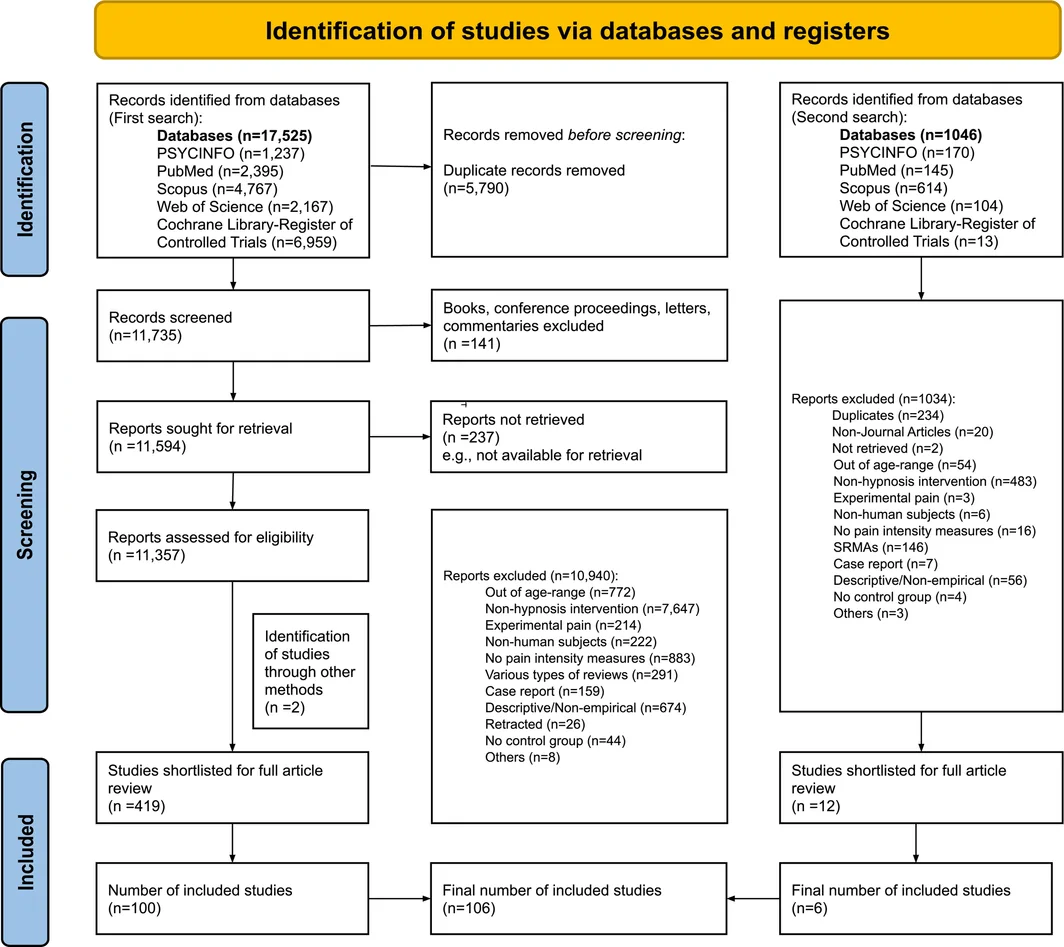
PRISMA flowchart.

The initial database search (18 November 2024) identified 17,525 records, with 5790 duplicates removed. We excluded 141 non‐journal articles and 237 records unavailable for retrieval, leaving 11,357 records for title/abstract screening. After applying eligibility criteria, 10,940 records were excluded (Figure 1). Two additional studies identified from recently published reviews were added, yielding 419 reports for full‐text review. Inter‐rater agreement at full text was moderate (Cohen's kappa = 0.58; SE = 0.04; 95% CI 0.51–0.66). One exception was made: Ter Kuile et al. (1994) was included as a hypnotic intervention despite being labelled ‘cognitive self‐hypnosis’, because the protocol primarily consisted of hypnosis‐related induction and strategies (e.g., relaxation, imaginative inattention and pain displacement/transformation) with limited cognitive restructuring. After consensus, 100 studies met criteria from the initial search.

The updated search (October 2025) identified 1046 additional records; 12 underwent full‐text screening and six were included. In total, 106 studies were included, from which 186 effect sizes were extracted. Overall, 104 effect sizes from 84 studies were pooled across meta‐analyses; the remaining 22 studies were primarily narratively synthesised (see Data S5). Study characteristics and findings are summarised in Tables 2 and 3.

**TABLE 2:** Characteristics of all 106 studies included in this review.

No.	Author/Year	Country	Total *N* (M, F)	Mean age (Range)	*N* (H)	*N* (C)	Pain type	Hypnosis modality	Control used^b^
1	Stam et al. (1986)	Denmark, Europe	41 (35F, 6M)	56 (NA)	22	22	C: Persistent idiopathic orofacial pain	IPT	RLX
2	Evans and Richardson (1988)^a^	UK, Europe	39 (39F, 0M)	42.82 (NA)	19	20	A: Abdominal Hysterectomy	SELF	PL
3	VanDyck et al. (1991)^a^	Netherlands, Europe	55 (28F, 27M)	36 (18–60)	27	28	C: Tension‐Type Headache	SELF	RLX(AT)
4	Liu et al. (1992)^a^	UK, Europe	73 (73F, 0M)	42.1 (NA)	24	25	A: Hysterectomy	IPT	PL
5	Patterson et al. (1992)^a^	USA, North America	30	34.1 (NA)	10	10	A: Burn Debridement	TRP	TAU/EDU
6	Spinhoven et al. (1992)^a^	Netherlands, Europe	46 (28F, 18M)	36 (20–62)	23	23	C: Chronic tension headache	TRP + SELF	RLX (AT)
7	Syrjala et al. (1992)^a^	USA, North America	45 (19F, 26M)	32.70 (19–49)	12	10–12	A: Bone Marrow Transplant	TRP + SELF	TAU/PL/CBT
8	Zitman et al. (1992)^a^	Netherlands, Europe	79 (43F, 36M)	34.80 (> 18)	24	28	C: Headache (> 6 months)	IPT	RLX(AT)
9	Everett et al. (1993)^a^	USA, North America	32 (3F, 29M)	37.30 (18–60)	8	8	A: Major burns	TRP	PL
10	Spanos et al. (1993)	Canada, North America	103 (82F, 31M)	NA (NA)	16	26	C: Chronic Migraine	IPT	PL
11	Ter Kuile et al. (1994)^a^	Netherlands, Europe	134 (85F, 49M)	NA (NA)	40	41–53	C: Recurrent headache	SELF	WL/RLX(AT)
12	Melzack et al. (1996)^a^	Canada, North America	20 (11F, 9M)	51.3 (24–73)	10	10	A: Colectomy or hysterectomy	IPT	EDU
13	Moore and Wiesner (1996)^a^	USA, North America	30 (28F, 2M)	70.8 (NA)	15	15	C: Upper Extremity Repetitive Strain Injuries	TRP + SELF	WL
14	Patterson and Ptacek (1997)^a^	USA, North America	61 (10F, 51M)	36.86 (NA)	31	30	A: Burn Treatment	TRP	PL
15	Ghoneim et al. (2000)^a^	USA, North America	60 (35F, 25M)	23.2 (18–35)	30	30	A: Third molar surgery	IPT	TAU
16	Wright and Drummond (2000)^a^	Australia	30 (8F, 22M)	34.80 (16–48)	15	15	A: Procedural pain for burns	TRP	TAU
17	Frenay et al. (2001)	Belgium, Europe	26 (13F, 13M)	40.98 (18–65)	11	15	A: Burn	TRP	SRS
18	Nilsson et al. (2001)	Sweden, Europe	90 (90F, 0M)	51 (NA)	31	30	A: Hysterectomy	IPT	MUS(ADD)
19	Gay et al. (2002)^a^	France, Europe	36 (33F, 3M)	64.7 (NA)	13	10–13	C: Hip or knee osteoarthritis	TRP	WL/RLX
20	Montgomery et al. (2002)^a^	USA, North America	20 (20F, 0M)	50.11 (30–81)	10	10	A: Excisional breast biopsy	TRP	TAU
21	Palsson et al. (2002)^a^	USA, North America	24 (15F, 9M)	39.1 (NA)	15	9	C: Irritable Bowel Syndrome	TRP + SELF	WL
22	Ginandes et al. (2003)	USA, North America	18 (18F, 0M)	38.9 (26–56)	6	4–6	A: Reduction mammaplasty	TRP + SELF	TAU/PL
23	Nilsson et al. (2003)	Sweden, Europe	182 (50F, 132M)	51.71 (NA)	57	62	A: Varicose vein or open inguinal hernia repair surgery	IPT	MUS(ADD)
24	Jones et al. (2006)^a^	UK, Europe	28 (10F, 18M)	57.21 (NA)	15	13	A: Non‐cardiac Chest Pain	TRP + SELF	PL
25	Roberts et al. (2006)	UK, Europe	81 (69F, 12M)	41.6 (18–65)	40	41	C: Irritable Bowel Syndrome	TRP + SELF	TAU
26	Askay et al. (2007)^a^	USA, North America	46	37 (NA)	27	19	A: Burn wound care	IPT	RLX
27	Castel et al. (2007)^a^	Spain, Europe	45 (39F, 6M)	43.7 (25–68)	15	15	C: Fibromyalgia	TRP	RLX
28	Montgomery et al. (2007)^a^	USA, North America	200 (200F, 0M)	48.5 (NA)	105	95	A: Lumpectomy or excisional breast biopsy	TRP	PL
29	Abrahamsen et al. (2008)	Denmark, Europe	41 (35F, 6M)	56 (NA)	22	19	C: Persistent idiopathic orofacial pain	IPT	RLX
30	Marc et al. (2008)^a^	Canada, North America	348 (348F, 0M)	25.2 (18–46)	172	175	A: First‐trimester surgical abortion	TRP	TAU
31	Shakibaei et al. (2008)^a^	Iran, Asia	44 (19F, 25M)	23.6 (6–62)	22	22	A: Burn	TRP	TAU
32	Abrahamsen et al. (2009)^a^	Denmark, Europe	40 (40F, 0M)	38.6 (NA)	20	19	C: Temporomandibular disorders	IPT	RLX
33	Castel et al. (2009)	Spain, Europe	39 (37F, 2M)	44.2 (18–60)	16	16	C: Fibromyalgia	TRP + SELF	CBT(ADD)
34	Jensen et al. (2009b)^a^	USA, North America	22 (16F, 6M)	51.7 (27–75)	15	7	C: Multiple sclerosis	TRP + SELF	RLX
35	Jensen et al. (2009a)^a^	USA, North America	37 (9F, 28M)	49.5 (19–70)	20	11	C: Spinal‐cord injury	TRP + SELF	BIO/RLX
36	Slack et al. (2009)^a^	USA, North America	26 (9F, 17M)	53.6 (30–78)	18	8	A: Needle electromyography	IPT	PL
37	Mackey (2010)	USA, North America	91 (54F, 37M)	NA (18–25)	46	54	A: Third molar surgery	IPT	MUS
38	Patterson et al. (2010)^a^	USA, North America	21 (4F, 17M)	31.8 (13–59)	12	9	A: Physical trauma	V	TAU/DIS^c^
39	Castel et al. (2012)	Spain, Europe	93 (90F, 3M)	49.64 (NA)	23–29	32–34	C: Fibromyalgia	TRP + SELF	CBT(ADD)
40	Lindfors et al. (2012)^a^	Sweden, Europe	48 (39F, 9M)	40 (22–60)	25	23	C: Irritable Bowel Syndrome	TRP	WL
41	Guittier et al. (2013)^a^	Switzerland, Europe	185 (185F, 0M)	32 (NA)	63	122	A: Childbirth	TRP	TAU
42	Phillips‐Moore et al. (2015)^a^	Australia	51 (44F, 7M)	40 (17–75)	17	17	C: Irritable Bowel Syndrome	TRP + SELF	RLX
43	Vanhaudenhuyse et al. (2015)^a^	Belgium, Europe	527 (440F, 87M)	54.83 (NA)	158	50–88	C: Chronic pain	TRP + SELF	WL/EDU/MSG
44	Akgul et al. (2016)^a^	Turkey	44 (9F, 35M)	54.5 (NA)	22	22	A: Coronary artery bypass grafting	TRP	PL
45	Ardigo et al. (2016)	Switzerland, Europe	53 (39F, 14M)	80.60 (NA)	17–23	17	C: Chronic pain	TRP + SELF	MSG
46	Peters et al. (2016)	Australia	74 (60F, 14M)	Median: 34–40 (NA)	25	24–25	C: Irritable Bowel Syndrome	TRP + SELF	DIET (ADD/COM)
47	Téllez et al. (2016)	Mexico, North America	75 (75F, 0M)	50 (NA)	24	20	A: Breast biopsy	IPT	MUS(ADD)
48	Beevi et al. (2017)^a^	Malaysia, Asia	45 (45F, 0M)	28.76 (NA)	23	22	A: Childbirth	TRP + SELF	TAU
49	Garland et al. (2017)^a^	USA, North America	244 (140F, 104M)	51.1 (18–93)	73	85–86	A: Acute pain	TRP	EDU/MF
50	Hosseinzadegan et al. (2017)^a^	Iran, Asia	60 (60F, 0M)	28.7 (NA)	30	30	C: Multiple sclerosis	TRP + SELF	TAU
51	Waisblat et al. (2017)^a^	France, Europe	155 (155F, 0M)	30.00 (NA)	79	76	A: Epidural catheter insertion for Childbirth	TRP	PL
52	Amraoui et al. (2018)^a^	France, Europe	148 (148F, 0M)	57 (33–79)	77	71	A: Minor breast cancer surgery	TRP	TAU
53	Jafarizadeh et al. (2018)^a^	Iran, Asia	60 (0F, 60M)	30.5 (NA)	30	30	A: Burn	TRP	PL
54	Jensen et al. (2018)	USA, North America	32 (24F, 8M)	57.53 (NA)	4–6	5	C: Multiple sclerosis	SELF	BIO(ADD)/MF(ADD)
55	Mackey (2018)	USA, North America	143	NA (NA)	46	54	A: Molar Extraction	IPT	MUS
56	Rizzo et al. (2018)	Brazil, South America	99 (80F, 19M)	50.05 (NA)	49	50	C: Chronic nonspecific low back pain	TRP	EDU(ADD)
57	Tastan et al. (2018)	Turkey	90 (64F, 26M)	33 (18–65)	27	30	C: Migraine	TRP	ACU
58	Lee et al. (2019)^a^	Malaysia, Asia	25 (23F, 2M)	63.25 (NA)	8	8	A: Total Knee Replacement	IPT	TAU
59	Paredes et al. (2019)^a^	Portugal, Europe	18 (0F, 18M)	45 (NA)	8	10	C: Haemophilia	TRP	TAU
60	Razak et al. (2019)	Malaysia, Asia	40 (0F, 40M)	35.83 (18–68)	20	20	C: Chronic brachial neuralgia	TRP + SELF	ACU
61	Sánchez‐Jáuregui et al. (2019)^a^	Mexico, North America	170 (170F, 0M)	47 (NA)	58	55–57	A: Breast Biopsy	IPT	TAU/MUS
62	Aravena et al. (2020)^a^	Chile, South America	97 (48F, 49M)	45 (NA)	48	47	C: Fibromyalgia	SELF	WL
63	Fusco et al. (2020)^a^	France, Europe	272 (150F, 122M)	54.5 (18–89)	89	91	A: Peripheral Intravenous Catheterization	TRP	PL
64	Garcia et al. (2020)^a^	France, Europe	113 (24F, 89M)	69.60 (NA)	56	57	A: Atrial flutter ablation	TRP	RLX
65	Jensen et al. (2020)^a^	USA, North America	173 (102F, 71M)	55.09 (> 18)	41–44	39–41	C: Chronic pain	TRP + SELF	CBT (COM/ADD)/EDU (COM/ADD)
66	Nowak et al. (2020)^a^	Germany, Europe	385 (225F, 160M)	NA (18–70)	191	194	A: Surgery	IPT	PL
67	Werner et al. (2020)^a^	Denmark, Europe	349 (349F, 0M)	29.88 (NA)	136	79–134	A: Childbirth	SELF	TAU/RLX
68	Bicego et al. (2021)^a^	Belgium, Europe	203 (182F, 21M)	46.7 (NA)	22–24	13–20	C: Chronic pain	TRP + SELF	CBT/SC(ADD)/MUS
69	Eaton et al. (2021)^a^	USA, North America	40 (31F, 9M)	52.7 (21–76)	21	19	C: Cancer survivor	SELF	WL
70	Hanley et al. (2021)^a^	USA, North America	285 (181F, 104M)	63.2 (NA)	89	90–106	A: Total knee arthroplasty	TRP	CBT/MF
71	Joo et al. (2021)^a^	Korea, Asia	38 (22F, 16M)	62.6 (NA)	19	19	A: Fluoroscopy‐guided minimally‐invasive intervention	V	TAU
72	Lang et al. (2021)^a^	USA, North America	72 (50F, 22M)	46.3 (18–78)	37	35	C: Craniofacial pain	SELF	PL
73	Paredes et al. (2021)^a^	Portugal, Europe	18 (0F, 18M)	45 (NA)	8	10	C: Haemophilia	TRP + SELF	TAU
74	Tezcan et al. (2020)^a^	Turkey	90 (0F, 90M)	64.2 (52–77)	45	45	A: Rigid cystoscopy	TRP	TAU
75	Tonye‐Geoffroy et al. (2021)	France, Europe	70 (17F, 53M)	49.1 (18–80)	36	34	C: Chronic non‐cancer pain	TRP	TENS(ADD)
76	Askay et al. (2022)^a^	USA, North America	153 (44F, 109M)	34 (NA)	73	36–43	A: Traumatic injury	V	PL(DIS)/TAU
77	Brooks et al. (2022)^a^	Australia	20 (20F, 0M)	34 (NA)	7	7	C: Persistent Pelvic Pain	SELF	WL
78	Coulibaly et al. (2022)^a^	France, Europe	86 (20F, 66M)	66 (NA)	25	61	A: Electrophysiological procedures	V	TAU
79	Lemoine et al. (2022)^a^	France, Europe	160 (160F, 0M)	61.4 (NA)	78	82	A: Marker placement procedure for Breast Cancer	TRP	TAU
80	Mendoza et al. (2022)^a^	USA, North America	152 (64F, 88M)	55.54 (NA)	38	39	C: Multiple sclerosis, spinal‐cord injury, acquired amputation or muscular dystrophy	TRP + SELF	CBT (COM/ADD)/EDU
81	Moreno Hernández et al. (2022)^a^	Mexico, North America	40 (40F, 0M)	54.13 (NA)	20	20	A: Breast Cancer Mastectomy	IPT	TAU
82	Portel et al. (2022)^a^	France, Europe	60 (19F, 41M)	63.5 (53–72)	28	30	A: Flexible Bronchoscopy	TRP	TAU
83	Williams et al. (2022)^a^	USA, North America	328 (85F, 243M)	53.16 (NA)	110	108–110	C: Chronic Pain	TRP + SELF	EDU/MF
84	Eaton et al. (2023)^a^	USA, North America	109 (89F, 20M)	55.64 (21.4–78.4)	49	46	C: Cancer	SELF	RLX
85	Gullo et al. (2023)^a^	Switzerland, Europe	100 (50F, 50M)	47.4 (18–84)	50	50	A: Endovascular interventions	V	TAU
86	Jensen et al. (2023)^a^	USA, North America	173 (102F, 71M)	55.76 (NA)	43–44	42–44	C: Chronic Pain (Multiple Sclerosis, Muscular Dystrophy, Spinal Cord Injury, Acquired amputation)	TRP + SELF	EDU/CBT (COM/ADD)
87	Kaczmarska et al. (2023)^a^	Poland, Europe	60 (43F, 17M)	32.8 (NA)	29	31	C: Chronic nociplastic pain	IPT	PL
88	Khazraee et al. (2023)^a^	Iran, Asia	38 (38F, 0M)	35.12 (NA)	19	19	C: Chronic Migraine	TRP + SELF	TAU
89	Rougereau et al. (2023)^a^	France, Europe	60 (56F, 4M)	55 (NA)	30	30	A: Ambulatory Hallux Valgus Surgery	V	TAU
90	Shahriyaripoor et al. (2023)^a^	Iran, Asia	22 (22F, 0M)	NA (18–45)	10	10	C: Chronic Pelvic Pain (Endometriosis)	TRP + SELF	TAU
91	Anastas et al. (2024)^a^	USA, North America	68 (14F, 52M, 2NB)	NA (NA)	23	15–20	C: Chronic Pain	TRP + SELF	EDU/MF
92	Rosenbloom et al. (2024)	Canada, North America	92 (49F, 43M)	57.6 (NA)	39	45	A: Major Oncologic Surgery	TRP + SELF	TAU
93	Azam et al. (2024)^a^	USA, North America	57 (28F, 29M)	55.84 (18+)	29	28	A: Oncological Surgery	TRP + SELF	TAU
94	Barat et al. (2025)^a^	France, Europe	70 (33F, 37M)	55.07 (19–81)	34	36	A: Percutaneous Liver Biopsy	TRP	TAU
95	Chiuvé et al. (2024)	Switzerland, Europe	19 (6F, 13M)	63 (NA)	10	9	A: Stereotactic Frame Fixation in Deep Brain Stimulation	TRP	TAU
96	Dorta et al. (2024)^a^	Brazil, South America	49 (28F, 21M)	45.36 (NA)	24	25	C: Fibromyalgia	TRP + SELF	PL
97	Monolo et al. (2024)	Italy, Europe	50 (28F, 22M)	64.82 (NA)	25	25	A: Peripherally Inserted Central Venous Catheter placement	TRP	TAU
98	Ozgunay et al. (2024)	Turkey	47 (47F, 0M)	27.85 (26–63)	24	23	C: Fibromyalgia	TRP + SELF	TAU
99	Anderson et al. (2025)	Australia	240 (217F, 23M)	NA (20–65)	121	119	C: Irritable Bowel Syndrome	SELF	RLX
100	Starosta et al. (2024)	USA, North America	51 (18F, 31M, 2NB)	50.9 (NA)	28	23	C: Spinal‐cord injury	TRP + SELF	TAU
101	Aravena et al. (2025)^a^	Chile, South America	97 (95F, 2M)	NA (NA)	48	49	C: Fibromyalgia	IPT	WL
102	Haidamous et al. (2025)^a^	USA, North America	49 (23F, 26M)	71 (NA)	25	24	A: Shoulder Replacement Surgery	IPT	TAU
103	Jensen et al. (2025)^a^	USA, North America	54 (42F, 12M)	56.2 (NA)	27	27	C: Chronic pain (Low back pain, Spinal Cord Injury, Multiple Sclerosis)	SELF	WL
104	Maamar et al. (2025)^a^	France, Europe	78 (38F, 40M)	60.6 (NA)	39	39	A: Unplanned procedural pain	TRP	TAU
105	Moretti et al. (2025)^a^	Italy, Europe	42 (34F, 8M)	47.3 (NA)	21	21	A: Bariatric Surgery	TRP	TAU
106	Rhodes et al. (2025)^a^	USA, North America	181	NA (NA)	60	61	A: Orthopaedic Surgery	V/IPT	TAU

Abbreviations: A, acute pain; ACU, acupuncture; ADD, addition of stated intervention on top of hypnosis; AT, autogenic training; BIO, bio‐feedback relaxation; C, chronic; CBT, cognitive‐behavioural therapy; COM, comparison between hypnosis and stated control intervention; DIS, distraction; EDU, pain education; F, female; H, hypnosis; IPT, in‐person taped/audio hypnosis; M, male; MF, mindfulness‐based intervention; MSG, massage; MUS, music; NB, non‐binary; PHY, physiotherapy; PL, placebo; RLX, relaxation; SELF, self‐hypnosis only; SRS, stress‐reducing strategies; TAU, treatment as usual; TENS, transcutaneous electrical nerve stimulation; TRP, in‐person therapist‐led hypnosis; TRP + SELF, in‐person therapist with regular self‐hypnosis; V, virtual‐reality hypnosis; WL, waiting list.

^a^ Indicates studies that have been included in at least one meta‐analysis.

^b^ Unless otherwise stated (i.e., when ‘ADD’ is stated), all controls did not concurrently introduce hypnotic suggestions and were compared directly to hypnosis.

^c^ Due to experimental design considerations, Patterson et al. (2010) combined the distraction group with the treatment‐as‐usual group to form a single control group.

**TABLE 3:** Study findings of all included 106 studies in this review.

No.	Author/Year	Pain type	*N* (H)	*N* (C)	Hypnosis modality	Hypnosis modality
1	Stam et al. (1986)	C: Persistent idiopathic orofacial pain	22	22	IPT	COM/P (↓ HYP vs. RLX): −1.3 (1.88) vs. −0.1 (0.87)^b^
2	Evans and Richardson (1988)^a^	A: Abdominal Hysterectomy	19	20	SELF	COM/P (↓ HYP vs. PL): 2.39 (2) vs. 2.65 (2.54); *p* = ns
3	VanDyck et al. (1991)^a^	C: Tension‐Type Headache	27	28	SELF	COM/P (↓ HYP vs. AT): 2 (2.01) vs. 2.02 (1.28); *p* < 0.01
4	Liu et al. (1992)^a^	A: Hysterectomy	24	25	IPT	COM/P (HYP vs. PL ↓): 3.97 (2.06) vs. 3.59 (1.45); *p* = ns
5	Patterson et al. (1992)^a^	A: Burn Debridement	10	10	TRP	COM/P (↓ HYP vs. EDU): 4.5 (2.72) vs. 6.6 (2.32); *p* = 0.03 COM/P (↓ HYP vs. TAU): 4.5 (2.72) vs. 6.9 (2.56); *p* = 0.01
6	Spinhoven et al. (1992)^a^	C: Chronic tension headache	23	23	TRP + SELF	COM/P (HYP vs. RLX/AT ↓): 3 (2.1) vs. 2.5 (1.4)
7	Syrjala et al. (1992)^a^	A: Bone Marrow Transplant	12	10–12	TRP + SELF	COM/P (↓ HYP vs. PL): 3.06 (2.07) vs. 4.61 (2.48); *p* = 0.031 COM/P (↓ HYP vs. CBT): 3.06 (2.07) vs. 4.92 (2.26); *p* = 0.031
8	Zitman et al. (1992)^a^	C: Headache (> 6 months)	24	28	IPT	COM/P (HYP vs. RLX/AT ↓): 5.29 (4.87) vs. 4.86 (3.06)
9	Everett et al. (1993)^a^	A: Major burns	8	8	TRP	COM/P (↓ HYP vs. PL): 3.7 (1.42) vs. 4.6 (1.6)
10	Spanos et al. (1993)	C: Chronic Migraine	16	26	IPT	COM/FUL1 (HYP vs. PL ↓): 1.69 (1.71) vs. 1.58 (1.06); *p* = ns
11	Ter Kuile et al. (1994)^a^	C: Recurrent headache	40	41–53	SELF	COM/P (HYP vs. RLX/AT ↓): 4.5 (2.96) vs. 3.24 (2.42) COM/P (↓ HYP vs. WL): 4.5 (2.96) vs. 5.08 (3.2)
12	Melzack et al. (1996)^a^	A: Colectomy or hysterectomy	10	10	IPT	COM/P (HYP vs. EDU ↓): 6.6 (2.5) vs. 4.9 (2.7)
13	Moore and Wiesner (1996)^a^	C: Upper Extremity Repetitive Strain Injuries	15	15	TRP + SELF	COM/P (↓ HYP vs. WL): −5.68 (1.62) vs. −2.13 (2.49); *p* < 0.0001^b^
14	Patterson and Ptacek (1997)^a^	A: Burn Treatment	31	30	TRP	COM/P (↓ HYP vs. PL): 4.85 (2.39) vs. 6.26 (2.37); *p* = 0.09 (ns)
15	Ghoneim et al. (2000)^a^	A: Third molar surgery	30	30	IPT	COM/P (HYP vs. TAU ↓): 3.63 (1.83) vs. 3.11 (1.78)
16	Wright and Drummond (2000)^a^	A: Procedural pain for burns	15	15	TRP	COM/P (HYP vs. TAU ↓): 3.5 (1.55) vs. 2.8 (1.16)
17	Frenay et al. (2001)	A: Burn	11	15	TRP	COM/P (↓ HYP vs. SRS): 3.2 (3.1) vs. 4.1 (3.1)
18	Nilsson et al. (2001)	A: Hysterectomy	31	30	IPT	ADJ/P (HYP vs. MUS ↓): 2.3 (1.2) vs. 1.8 (0.7); *p* = ns
19	Gay et al. (2002)^a^	C: Hip or knee osteoarthritis	13	10–13	TRP	COM/P (↓ HYP vs. WL): 1.97 (1.31) vs. 5.05 (1.79) COM/P (↓ HYP vs. RLX): 1.97 (1.31) vs. 3.76 (1.77) COM/FUL3 (↓ HYP vs. WL): 1.66 (1.49) vs. 4.29 (1.31) COM/FUL3 (↓ HYP vs. RLX): 1.66 (1.49) vs. 2.75 (1.91)
20	Montgomery et al. (2002)^a^	A: Excisional breast biopsy	10	10	TRP	COM/P (↓ HYP vs. TAU): 1.41 (4.27) vs. 1.96 (1.15)
21	Palsson et al. (2002)^a^	C: Irritable Bowel Syndrome	15	9	TRP + SELF	COM/P (↓ HYP vs. WL): 2.3 (2.21) vs. 3 (0.64); *p* = 0.049
22	Ginandes et al. (2003)	A: Reduction mammaplasty	6	4–6	TRP + SELF	COM/FUL1W (HYP vs. TAU ↓): 3.17 (0.52) vs. 2.25 (2.63) COM/FUL3 (↓ HYP vs. TAU): 0.33 (0.82) vs. 1.83 (3.06) COM/FUL1W (↓ HYP vs. PL): 3.17 (0.52) vs. 3.83 (2.23) COM/FUL3 (↓ HYP vs. PL): 0.33 (0.82) vs. 1.67 (3.14)
23	Nilsson et al. (2003)	A: Varicose vein or open inguinal hernia repair surgery	57	62	IPT	ADJ/P (↓ HYP vs. MUS): 1.9 (1.5) vs. 2.1 (1.4); *p* = ns
24	Jones et al. (2006)^a^	A: Non‐cardiac Chest Pain	15	13	TRP + SELF	COM/P (↓ HYP vs. PL): 2.91 (2.53) vs. 4.73 (2.65); *p* = 0.046 COM/P (↓ HYP vs. PL): −3.05 (2.42) vs. −1.33 (1.84); *p* = 0.046^b^
25	Roberts et al. (2006)	C: Irritable Bowel Syndrome	40	41	TRP + SELF	COM/FUL3 (↓HYP vs. TAU): −2.12 (2.15) vs. −0.68 (2.56); *p* = 0.02^b^ COM/FUL12 (↓ HYP vs. TAU): −1.63 (2.59) vs. −1.57 (3.2); *p* = 0.76 (ns)^b^
26	Askay et al. (2007)^a^	A: Burn wound care	27	19	IPT	COM/P (HYP vs. RLX): 3.54 (2.19) vs. 3.54 (2.31)
27	Castel et al. (2007)^a^	C: Fibromyalgia	15	15	TRP	COM/P (↓ HYP vs. RLX): 1.7 (1.68) vs. 3.3 (2.58)
28	Montgomery et al. (2007)^a^	A: Lumpectomy or excisional breast biopsy	105	95	TRP	COM/P (↓ HYP vs. PL): 2.24 (4.78) vs. 2.54 (3.08)
29	Abrahamsen et al. (2008)	C: Persistent idiopathic orofacial pain	22	19	IPT	COM/P (↓ HYP vs. RLX): −1.3 (1.88) vs. −0.1 (0.94)^b^
30	Marc et al. (2008)^a^	A: First‐trimester surgical abortion	172	175	TRP	COM/P (↓ HYP vs. TAU): 0.99 (1.7) vs. 1.36 (1.9)
31	Shakibaei et al. (2008)^a^	A: Burn	22	22	TRP	COM/P (↓ HYP vs. TAU): 4.2 (2.9) vs. 6.6 (1.66); *p* = 0.002
32	Abrahamsen et al. (2009)^a^	C: Temporomandibular disorders	20	19	IPT	COM/P (↓ HYP vs. RLX): 2.9 (2.4) vs. 3.9 (1.5)
33	Castel et al. (2009)	C: Fibromyalgia	16	16	TRP + SELF	ADJ/P (↓ HYP vs. CBT): 5.79 (2.05) vs. 6.1 (2.52)
34	Jensen et al. (2009b)^a^	C: Multiple sclerosis	15	7	TRP + SELF	COM/P (↓ HYP vs. RLX): 1.32 (1.28) vs. 2.08 (1.58); *p* = 0.0001
35	Jensen et al. (2009a)^a^	C: Spinal‐cord injury	20	11	TRP + SELF	COM/P (HYP vs. BIO/RLX ↓): 5.09 (1.92) vs. 3.36 (1.28) COM/FUL3 (HYP vs. BIO/RLX ↓): 4.9 (2.13) vs. 3.13 (1.42)
36	Slack et al. (2009)^a^	A: Needle electromyography	18	8	IPT	COM/P (↓ HYP vs. PL): 2.6 (1.8) vs. 3.5 (2.6)
37	Mackey (2010)	A: Third molar surgery	46	54	IPT	COM/P (↓ HYP vs. MUS): 2.57 (1.48) vs. 3.97 (1.45); *p* < 0.0001
38	Patterson et al. (2010)^a^	A: Physical trauma	12	9	V	COM/P (↓ HYP vs. PL): 3.83 (2.86) vs. 4.78 (2.58)
39	Castel et al. (2012)	C: Fibromyalgia	23–29	32–34	TRP + SELF	ADJ/P (↓ HYP vs. CBT): 5.3 (0.3) vs. 5.6 (0.3) ADJ/FUL3 (↓ HYP vs. CBT): 5.7 (0.4) vs. 5.9 (0.3) ADJ/FUL6 (↓ HYP vs. CBT): 5.6 (0.4) vs. 5.7 (0.4)
40	Lindfors et al. (2012)^a^	C: Irritable Bowel Syndrome	25	23	TRP	COM/FUL3 (HYP vs. WL ↓): 6.14 (2) vs. 6 (2.43) COM/FUL3 (↓ HYP vs. WL): −1.14 (1.77) vs. −0.84 (2.11); *p* = ns^b^
41	Guittier et al. (2013)^a^	A: Childbirth	63	122	TRP	COM/P (↓ HYP vs. TAU): 5.58 (2.45) vs. 6.02 (2.45); *p* = 0.25 (ns)
42	Phillips‐Moore et al. (2015)^a^	C: Irritable Bowel Syndrome	17	17	TRP + SELF	COM/P (↓ HYP vs. RLX): 3.7 (2.6) vs. 4.3 (2.6)
43	Vanhaudenhuyse et al. (2015)^a^	C: Chronic pain	158	50–88	TRP + SELF	COM/P (↓ HYP vs. WL): 4.6 (2) vs. 5.7 (2.3) COM/P (↓ HYP vs. EDU): 4.6 (2) vs. 5.5 (2.3) COM/P (↓ HYP vs. MSG): 4.6 (2) vs. 5.3 (2.2)
44	Akgul et al. (2016)^a^	A: Coronary artery bypass grafting	22	22	TRP	COM/P (↓ HYP vs. PL): 0.5 (0.9) vs. 0.73 (1.1)
45	Ardigo et al. (2016)	C: Chronic pain	17–23	17	TRP + SELF	COM/P (↓ HYP vs. MSG): 2.6 (2.8) vs. 3.6 (2.6) COM/FUL3 (↓ HYP vs. MSG): 3 (2.8) vs. 3.6 (2.1)
46	Peters et al. (2016)	C: Irritable Bowel Syndrome	25	24–25	TRP + SELF	COM/FUL3 (↓ HYP vs. DIET): −2.7 (2.68) vs. −2.6 (3.12); *p* = ns^b^ COM/FUL6 (↓ HYP vs. DIET): −3.3 (3.32) vs. −3 (2.62); *p* = ns^b^ ADJ/FUL3 (↓ HYP vs. DIET): −3.1 (2.81) vs. −2.7 (2.68); *p* = ns^b^ ADJ/FUL6 (HYP vs. DIET ↓): −2.9 (3.19) vs. −3.3 (3.32); *p* = ns^b^
47	Téllez et al. (2016)	A: Breast biopsy	24	20	IPT	ADJ/P (IPT vs. MUS ↓): −1.3 (1.9) vs. −1.7 (2.4); *p* = ns^b^
48	Beevi et al. (2017)^a^	A: Childbirth	23	22	TRP + SELF	COM/P (HYP vs. TAU ↓): 5.49 (2.48) vs. 3.42 (1.9); *p* = 0.004
49	Garland et al. (2017)^a^	A: Acute pain	73	85–86	TRP	COM/P (↓ HYP vs. EDU): 4.38 (2.9) vs. 4.67 (2.84); *p* < 0.001 COM/P (↓ HYP vs. MF): 4.38 (2.9) vs. 4.56 (2.79); *p* = ns
50	Hosseinzadegan et al. (2017)^a^	C: Multiple sclerosis	30	30	TRP + SELF	COM/P (↓ HYP vs. TAU): 3.7 (1.7) vs. 5.5 (5.4); *p* < 0.005 COM/FUL1 (↓ HYP vs. TAU): 4.9 (0.9) vs. 5.4 (1.5)
51	Waisblat et al. (2017)^a^	A: Epidural catheter insertion for Childbirth	79	76	TRP	COM/P (↓ HYP vs. PL): 4.9 (1.59) vs. 5.8 (2.22); *p* = 0.004
52	Amraoui et al. (2018)^a^	A: Minor breast cancer surgery	77	71	TRP	COM/P (HYP vs. TAU ↓): 2.63 (1.62) vs. 1.75 (1.59)
53	Jafarizadeh et al. (2018)^a^	A: Burn	30	30	TRP	COM/P (↓ HYP vs. PL): 5.02 (1.53) vs. 5.33 (1.86)
54	Jensen et al. (2018)	C: Multiple sclerosis	4–6	5	SELF	ADD/P (↓ HYP vs. BIO): 2.56 (1.2) vs. 4.5 (2.61) ADD/FUL1 (↓ HYP vs. BIO): 2.42 (1.23) vs. 4.48 (2.17) ADD/P (↓ HYP vs. MF): 2.3 (2.42) vs. 4.5 (2.61) ADD/FUL1 (↓ HYP vs. MF): 3.31 (1.28) vs. 4.48 (2.17)
55	Mackey (2018)	A: Molar Extraction	46	54	IPT	COM/P (↓ HYP vs. MUS): 1.48 (2.57) vs. 3.97 (1.45); *p* < 0.0001
56	Rizzo et al. (2018)	C: Chronic nonspecific low back pain	49	50	TRP	ADJ/P (↓ HYP vs. EDU): 4.4 (2.14) vs. 5.6 (2.21) ADJ/FUL3 (↓ HYP vs. EDU): 4.4 (2.93) vs. 5.1 (2.48)
57	Tastan et al. (2018)	C: Migraine	27	30	TRP	COM/P (↓ HYP vs. ACU): 1.2 (0.8) vs. 4 (1.6)
58	Lee et al. (2019)^a^	A: Total Knee Replacement	8	8	IPT	COM/P (↓ HYP vs. TAU): 1.77 (0.83) vs. 2.59 (1.47); *p* = 0.316 (ns) COM/FUL1 (HYP vs. TAU ↓): 3.7 (1.54) vs. 3.33 (1.07) COM/FUL3 (↓ HYP vs. TAU): 2.43 (1.79) vs. 2.45 (0.56) COM/FUL6 (↓ HYP vs. TAU): 1.4 (0.89) vs. 2.23 (1.41)
59	Paredes et al. (2019)^a^	C: Haemophilia	8	10	TRP	COM/P (HYP vs. TAU ↓): 4.13 (1.09) vs. 3.81 (1.51) COM/P (HYP vs. TAU ↓): −0.09 (1.48) vs. −0.46 (2.11); *p* = 0.686 (ns)^b^
60	Razak et al. (2019)	C: Chronic brachial neuralgia	20	20	TRP + SELF	COM/P (HYP vs. ACU ↓): 3.1 (1.25) vs. 2.85 (1.31)^b^ COM/FUL6 (HYP vs. ACU ↓): 2.2 (1.11) vs. 1.35 (0.81)^b^ COM/P (HYP vs. ACU ↓): 4.95 (1.76) vs. 4.4 (2.19) COM/FUL6 (HYP vs. ACU ↓): 5.9 (1.37) vs. 5.85 (1.39)
61	Sánchez‐Jáuregui et al. (2019)^a^	A: Breast Biopsy	58	55–57	IPT	COM/P (↓ HYP vs. TAU): 2.5 (3.2) vs. 3.2 (3.4); *p* = 0.21 (ns) COM/P (HYP vs. MUS ↓): 2.5 (3.2) vs. 2 (2.8); *p* = 0.18 (ns)
62	Aravena et al. (2020)^a^	C: Fibromyalgia	48	47	SELF	COM/P (↓ HYP vs. WL): 4.75 (1.54) vs. 5.92 (1.43); *p* < 0.001
63	Fusco et al. (2020)^a^	A: Peripheral Intravenous Catheterization	89	91	TRP	COM/P (↓ HYP vs. PL): 1.5 (1.9) vs. 3.5 (2.3); *p* < 0.0001
64	Garcia et al. (2020)^a^	A: Atrial flutter ablation	56	57	TRP	COM/P (↓ HYP vs. RLX): 4 (2.2) vs. 5.5 (1.8)
65	Jensen et al. (2020)^a^	C: Chronic pain	41–44	39–41	TRP + SELF	COM/P (↓ HYP vs. EDU): −0.78 (1.12) vs. −0.76 (1.26)^b^ COM/P (HYP vs. CBT ↓): −0.78 (1.12) vs. −1.32 (1.73)^b^ ADJ/P (↓ HYP vs. CBT): −1.54 (1.93) vs. −1.32 (1.73)^b^ COM/P (↓ HYP vs. EDU): 3.67 (1.8) vs. 3.72 (1.91) COM/P (HYP vs. CBT ↓): 3.67 (1.8) vs. 3.58 (1.86) ADJ/P (↓ HYP vs. CBT): 3.37 (2.07) vs. 3.58 (1.86) COM/FUL6 (HYP vs. EDU ↓): 3.88 (2.09) vs. 3.66 (1.79) COM/FUL6 (HYP vs. CBT ↓): 3.88 (2.09) vs. 3.65 (1.79) ADJ/FUL6 (↓ HYP vs. CBT): 3.35 (2.08) vs. 3.65 (1.79)
66	Nowak et al. (2020)^a^	A: Surgery	191	194	IPT	COM/P (↓ HYP vs. PL): 1.4 (2.2) vs. 2.2 (2.7)
67	Werner et al. (2020)^a^	A: Childbirth	136	79–134	SELF	COM/P (↓ HYP vs. TAU): 6.7 (2.9) vs. 7.2 (2.5) COM/P (↓ HYP vs. RLX): 6.7 (2.9) vs. 7.1 (2.5)
68	Bicego et al. (2021)^a^	C: Chronic pain	22–24	13–20	TRP + SELF	COM/P (HYP vs. CBT): 5.88 (1.62) vs. 5.88 (1.65) COM/FUL6 (↓ HYP vs. CBT): 5.57 (1.91) vs. 6.11 (1.52) COM/P (↓ HYP vs. MUS): 5.88 (1.62) vs. 5.91 (1.45); *p* = ns COM/FUL6 (↓ HYP vs. MUS): 5.75 (1.91) vs. 5.83 (1.44) ADJ/P (HYP vs. SC ↓): 5.88 (1.62) vs. 5.42 (1.66) ADJ/FUL6 (HYP vs. SC ↓): 5.75 (1.91) vs. 5.51 (1.82)
69	Eaton et al. (2021)^a^	C: Cancer survivor	21	19	SELF	COM/P (↓ HYP vs. WL): 6.15 (2.18) vs. 6.61 (1.38)
70	Hanley et al. (2021)^a^	A: Total knee arthroplasty	89	90–106	TRP	COM/P (↓ HYP vs. CBT): 3.45 (1.52) vs. 4.11 (1.72); *p* = significant COM/P (HYP vs. MF ↓): 3.45 (1.52) vs. 3.38 (2); *p* = ns
71	Joo et al. (2021)^a^	A: Fluoroscopy‐guided minimally‐invasive intervention	19	19	V	COM/P (↓ HYP vs. TAU): 3.7 (1.4) vs. 5.5 (1.7); *p* = 0.002
72	Lang et al. (2021)^a^	C: Craniofacial pain	37	35	SELF	COM/P (HYP vs. PL ↓): 2.59 (2.03) vs. 2.51 (2.17) COM/P (↓ HYP vs. PL): −0.76 (2.34) vs. −0.25 (0.92)^b^
73	Paredes et al. (2021)^a^	C: Haemophilia	8	10	TRP + SELF	COM/P (HYP vs. TAU ↓): 4.13 (1.09) vs. 3.81 (1.51); *p* = ns COM/FUL3 (↓ HYP vs. TAU): 3.63 (2.15) vs. 4.07 (1.41); *p* = ns
74	Tezcan et al. (2020)^a^	A: Rigid cystoscopy	45	45	TRP	COM/P (↓ HYP vs. TAU): 7.7 (1.5) vs. 8.4 (1.2)
75	Tonye‐Geoffroy et al. (2021)	C: Chronic non‐cancer pain	36	34	TRP	ADJ/P (↓ HYP vs. TENS): −2.36 (2.25) vs. −2.26 (2.04); *p* = ns^b^ ADJ/P (↓ HYP vs. TENS): 3.43 (2.13) vs. 3.49 (2.03); *p* = ns
76	Askay et al. (2022)^a^	A: Traumatic injury	73	36–43	V	COM/P (↓ HYP vs. PL): 4.52 (2.21) vs. 5.1 (2.08) COM/P (HYP vs. TAU ↓): 4.52 (2.21) vs. 4.44 (1.95)
77	Brooks et al. (2022)^a^	C: Persistent Pelvic Pain	7	7	SELF	COM/P (↓ HYP vs. WL): 4.55 (15.9) vs. 4.68 (23.5); *p* = 0.907 (ns)
78	Coulibaly et al. (2022)^a^	A: Electrophysiological procedures	25	61	V	COM/P (HYP vs. TAU ↓): 3.74 (2.21) vs. 3.07 (2.89); *p* = ns
79	Lemoine et al. (2022)^a^	A: Marker placement procedure for Breast Cancer	78	82	TRP	COM/P (HYP vs. TAU ↓): 1.9 (2.3) vs. 1.6 (2.1); *p* = 0.825 (ns) COM/P (HYP vs. TAU ↓): 0.9 (2.7) vs. 0.8 (2.1)^b^
80	Mendoza et al. (2022)^a^	C: Multiple sclerosis, spinal‐cord injury, acquired amputation or muscular dystrophy	38	39	TRP + SELF	COM/P (↓ HYP vs. EDU): 2.54 (1.21) vs. 3.92 (2.13) COM/P (↓ HYP vs. EDU): −1.58 (1.74) vs. −0.18 (1)^b^ COM/P (↓ HYP vs. CBT): 2.54 (1.21) vs. 3.65 (2.43) COM/P (↓ HYP vs. CBT): −1.58 (1.74) vs. −0.7 (1.41)^b^ ADJ/P (↓ HYP vs. CBT): 2.69 (2.09) vs. 3.65 (2.43) ADJ/P (↓ HYP vs. CBT): −1.55 (1.77) vs. −0.7 (1.41)^b^
81	Moreno Hernández et al. (2022)^a^	A: Breast Cancer Mastectomy	20	20	IPT	COM/P (↓ HYP vs. TAU): −1.5 (1.54) vs. −1.25 (1.92); *p* = 0.360 (ns)^b^ COM/FUL1W (↓ HYP vs. TAU): −1.75 (2.42) vs. −0.4 (2.26); *p* = 0.07^b^
82	Portel et al. (2022)^a^	A: Flexible Bronchoscopy	28	30	TRP	COM/P (↓ HYP vs. TAU): 2.4 (2.8) vs. 3.6 (2.8)
83	Williams et al. (2022)^a^	C: Chronic Pain	110	108–110	TRP + SELF	COM/P (↓ HYP vs. EDU): −0.61 (0.29) vs. −0.57 (0.29); *p* = 0.39 (ns)^b^ COM/FUL3 (↓HYP vs. EDU): −0.95 (0.37) vs. −0.48 (0.35); *p* = 0.05^b^ COM/FUL6 (↓HYP vs. EDU): −1.04 (0.35) vs. −0.28 (0.32); *p* < 0.001^b^ COM/P (HYP vs. MF ↓): −0.61 (0.29) vs. −0.85 (0.29); *p* = 0.39 (ns)^b^ COM/FUL3 (↓HYP vs. MF): −0.95 (0.37) vs. −0.67 (0.34); *p* = 0.05^b^ COM/FUL6 (↓HYP vs. MF): −1.04 (0.35) vs. −0.86 (0.32); *p* < 0.001^b^
84	Eaton et al. (2023)^a^	C: Cancer	49	46	SELF	COM/P (↓ HYP vs. RLX): 4.49 (2.11) vs. 4.5 (1.95) COM/P (↓ HYP vs. RLX): −1.61 (2) vs. −1.35 (1.69)^b^
85	Gullo et al. (2023)^a^	A: Endovascular interventions	50	50	V	COM/P (↓ HYP vs. TAU): 2.04 (1.82) vs. 2.62 (2.42)
86	Jensen et al. (2023)^a^	C: Chronic Pain (Multiple Sclerosis, Muscular Dystrophy, Spinal Cord Injury, Acquired amputation)	43–44	42–44	TRP + SELF	COM/P (↓ HYP vs. EDU): −0.78 (1.12) vs. −0.76 (1.26)^b^ COM/P (HYP vs. CBT ↓): −0.78 (1.12) vs. −1.32 (1.73)^b^ ADJ/P (↓ HYP vs. CBT): −1.54 (1.93) vs. −1.32 (1.73)^b^
87	Kaczmarska et al. (2023)^a^	C: Chronic nociplastic pain	29	31	IPT	COM/P (HYP vs. PL ↓): 1.93 (2) vs. 1.74 (2.04)
88	Khazraee et al. (2023)^a^	C: Chronic Migraine	19	19	TRP + SELF	COM/P (↓ HYP vs. TAU): 2.77 (2.04) vs. 6.97 (1.3); *p* < 0.001
89	Rougereau et al. (2023)^a^	A: Ambulatory Hallux Valgus Surgery	30	30	V	COM/P (↓ HYP vs. TAU): 4 (2.4) vs. 5.1 (2.9); *p* = 0.09
90	Shahriyaripoor et al. (2023)^a^	C: Chronic Pelvic Pain (Endometriosis)	10	10	TRP + SELF	COM/P (HYP vs. TAU ↓): 1.8 (2.25) vs. 1.3 (1.42); *p* = ns COM/FUL1 (HYP vs. TAU ↓): 1.6 (2.01) vs. 1.1 (1.1); *p* = ns
91	Anastas et al. (2024)^a^	C: Chronic Pain	23	15–20	TRP + SELF	COM/P (HYP vs. EDU ↓): 5.46 (2.01) vs. 4.68 (2.09); *p* = ns COM/P (HYP vs. EDU ↓): 0.17 (1.28) vs. −0.48 (2.39); *p* = ns^b^ COM/P (HYP vs. MF ↓): 5.46 (2.01) vs. 4.33 (2.19); *p* = ns COM/P (HYP vs. MF ↓): 0.17 (1.28) vs. −0.17 (1.55); *p* = ns^b^
92	Anderson et al. (2025)	C: Irritable Bowel Syndrome	121	119	SELF	ADD/P (↓ HYP vs. RLX): 4.46 (2.18) vs. 4.99 (2.39)
93	Azam et al. (2024)^a^	A: Oncological Surgery	29	28	TRP + SELF	COM/FUL1W (↓ HYP vs. TAU): 3.12 (2.28) vs. 3.42 (1.69); *p* = ns COM/FUL3 (↓ HYP vs. TAU): 2.5 (2.50) vs. 3.0 (2.06); *p* = ns
94	Barat et al. (2025)^a^	A: Percutaneous Liver Biopsy	34	36	TRP	COM/P (↓ HYP vs. TAU): 2.4 (1.9) vs. 4.4 (2.6); *p* = 0.001
95	Chiuvé et al. (2024)^a^	A: Stereotactic Frame Fixation in Deep Brain Stimulation	10	9	TRP	COM/P (↓ HYP vs. TAU): 5.6 (2.1) vs. 6.4 (1.2); *p* = 0.31 (ns) COM/FUL6 (↓ HYP vs. TAU): 5.3 (3.4) vs. 6.2 (2.5); *p* = 0.7 (ns)
96	Dorta et al. (2024)^a^	C: Fibromyalgia	24	25	TRP + SELF	COM/P (↓ HYP vs. PL): 4.4 (0.9) vs. 9 (1.3); *p* < 0.05 COM/FUL3 (↓ HYP vs. PL): 4.5 (1.2) vs. 9.1 (1); *p* < 0.05
97	Monolo et al. (2024)^a^	A: Peripherally Inserted Central Venous Catheter placement	25	25	TRP	COM/P (↓ HYP vs. TAU): 1.44 (2.06) vs. 1.6 (1.8); *p* = 0.7716 (ns)
98	Ozgunay et al. (2024)	C: Fibromyalgia	24	23	TRP + SELF	COM/FUL6 (↓ HYP vs. TAU): 4.75 (2.72) vs. 6.73 (1.93); *p* = 0.162 (ns)
99	Rosenbloom et al. (2024)	A: Major Oncologic Surgery	39	45	TRP + SELF	COM/FUL1W (↓ HYP vs. TAU): 2.73 (2.3) vs. 3.15 (2.4); *p* = 0.93 (ns)
100	Starosta et al. (2024)^a^	C: Spinal‐cord injury	28	23	TRP + SELF	COM/P (↓ HYP vs. TAU): 5.06 (2.32) vs. 5.67 (1.85) COM/P (↓ HYP vs. TAU): −1.06 (1.73) vs. −0.5 (1.46)^b^
101	Aravena et al. (2025)^a^	C: Fibromyalgia	48	49	IPT	COM/P (↓ HYP vs. WL): 4.75 (1.54) vs. 5.92 (1.43); *p* = 0.062
102	Haidamous et al. (2025)^a^	A: Shoulder Replacement Surgery	25	24	IPT	COM/P (↓ HYP vs. TAU): 3 (1.91) vs. 6 (2.25); *p* = 0.017
103	Jensen et al. (2025)^a^	C: Chronic pain (Low back pain, Spinal Cord Injury, Multiple Sclerosis)	27	27	SELF	COM/P (↓ HYP vs. WL): 4.17 (1.4) vs. 4.56 (1.92)
104	Maamar et al. (2025)^a^	A: Unplanned procedural pain	39	39	TRP	COM/P (HYP vs. TAU): 2.2 (2.9) vs. 2.2 (3); *p* = 0.89 (ns)
105	Moretti et al. (2025)^a^	A: Bariatric Surgery	21	21	TRP	COM/P (↓ HYP vs. TAU): 2.9 (2.45) vs. 5.8 (2.25); *p* = 0.0007
106	Rhodes et al. (2025)^a^	A: Lumbar Puncture for Physical trauma	60	61	V/IPT	COM/P (V vs. TAU ↓): 2.15 (18.81) vs. 0.32 (13.97); *p* = 0.71 (ns)^b^ COM/P (↓ IPT vs. TAU): −1.97 (21.9) vs. 0.32 (13.97); *p* = 0.71 (ns)^b^

Abbreviations: A, acute pain; ACU, acupuncture; ADD, addition of stated intervention on top of hypnosis; ADJ, adjunctive use of hypnosis on top of stated intervention; AT, autogenic training; BIO, bio‐feedback relaxation; C, chronic; CBT, cognitive‐behavioural therapy; COM, comparison between hypnosis and stated control intervention; DIS, distraction; EDU, pain education; FUL1, follow‐up less than 1 month; FUL12, follow‐up 6–12 months; FUL1W, follow‐up less than 1 week; FUL3, follow‐up 1–3 months; FUL6, follow‐up 3–6 months; H, hypnosis; IPT, in‐person taped/audio hypnosis; MF, mindfulness‐based intervention; MSG, massage; MUS, music; NB, non‐binary; ns, not statistically significant; P, post‐intervention; PHY, physiotherapy; PL, placebo; RLX, relaxation; SELF, self‐hypnosis only; SRS, stress‐reducing strategies; TAU, treatment as usual; TENS, transcutaneous electrical nerve stimulation; TRP, in‐person therapist‐led hypnosis; TRP+SELF, in‐person therapist with regular self‐hypnosis; V, virtual‐reality hypnosis; WL, waiting list.

^a^ Indicates studies that have been included in at least one meta‐analyses.

^b^ Indicates pre‐post mean difference scores.

### 3.1 Sample Characteristics

Studies were conducted in 22 countries, most commonly the USA (34) and France (12), followed by Iran and Australia (five each); other countries contributed four or fewer studies. Across 106 studies, 9872 participants were included. Excluding four studies without gender reporting (Patterson et al. 1992; Mackey 2018; Askay et al. 2022; Rhodes et al. 2025), samples comprised 3000 (31.6%) men, 6478 (68.3%) women, and four participants identifying as non‐binary or transgender; female over‐representation was expected given higher chronic pain susceptibility in women (Bartley and Fillingim 2013). Among 65 studies reporting mean age, mean ages ranged from 23.2 years (Ghoneim et al. 2000) to 80.6 years (Ardigo et al. 2016). Fifty studies involved chronic pain conditions (e.g., fibromyalgia, diabetic neuropathic pain, cancer pain and head/neck pain) and 56 involved acute/procedural pain (e.g., elective surgery, burns, tooth extraction and prostate needle biopsy).

### 3.2 Hypnosis Intervention and Protocol

Delivery modality most commonly involved in‐person practitioner‐delivered hypnosis only (33 studies) or therapist‐delivered hypnosis supplemented by self‐hypnosis/self‐practice (31 studies). Thirteen studies primarily trained self‐hypnosis, 22 used in‐person tape/audio‐delivered hypnosis and seven used virtual reality. The mean number of sessions before outcome assessment was 4.81 (range: single perioperative session to 42 daily self‐hypnosis sessions; Anderson et al. 2025); session duration ranged from 10 min to 2 h. Many studies adapted established protocols, including Barber's Rapid Induction of Analgesia script (Barber 1977) (e.g., Everett et al. 1993; Abrahamsen et al. 2008; Slack et al. 2009; Jafarizadeh et al. 2018), Erickson and Rossi's indirect permissive approach (Erickson and Rossi 1979) (e.g., Gay et al. 2002; Akgul et al. 2016), or Torem's age progression/regression technique (Torem 1992) (e.g., Rizzo et al. 2018; Mendoza et al. 2022; Starosta et al. 2024). Across protocols, inductions typically involved attentional focus (e.g., eye fixation) and imaginative involvement; deepening commonly used progressive muscle relaxation (Frenay et al. 2001; Jafarizadeh et al. 2018), breathing exercises or hand levitation (Castel et al. 2009; Jafarizadeh et al. 2018). Analgesic suggestions included relaxation/well‐being, reduced pain intensity/unpleasantness, increased control or transformation/substitution of pain sensations (e.g., warmth, cooling and tingling), sometimes with post‐hypnotic suggestions to encourage durability (Eaton et al. 2023; Kaczmarska et al. 2024). Visualisation of a safe/quiet/preferred place was common (Stam et al. 1986; Abrahamsen et al. 2008; Abrahamsen et al. 2009; Jensen et al. 2009b; Jensen et al. 2009a; Ardigo et al. 2016; Amraoui et al. 2018; Garcia et al. 2020; Jensen et al. 2020; Bicego et al. 2021; Lang et al. 2021; Tonye‐Geoffroy et al. 2021), alongside other imagery such as a leaf floating (Castel et al. 2007) or an ‘analgesic glove’ (Bicego et al. 2021). Some studies incorporated self‐care (Vanhaudenhuyse et al. 2015), cognitive‐behavioural elements (Castel et al. 2009) or group formats (Castel et al. 2012).

### 3.3 Comparison Groups and Active Control Types

Relaxation was the most common active control (17 studies) (Stam et al. 1986; VanDyck et al. 1991; Spinhoven et al. 1992; Zitman et al. 1992; Ter Kuile et al. 1994; Gay et al. 2002; Askay et al. 2007; Castel et al. 2007; Abrahamsen et al. 2008; Abrahamsen et al. 2009; Jensen et al. 2009b; Jensen et al. 2009a; Phillips‐Moore et al. 2015; Garcia et al. 2020; Werner et al. 2020; Eaton et al. 2023; Anderson et al. 2025), typically incorporating body scanning and somatic suggestions to deepen relaxation; some used Jacobson's progressive muscle relaxation (Gay et al. 2002; Jensen et al. 2009b) or autogenic training (VanDyck et al. 1991; Spinhoven et al. 1992; Zitman et al. 1992; Ter Kuile et al. 1994). One study used biofeedback to enhance relaxation (Jensen et al. 2009b), and one blended relaxation with pain education and cognitive behavioural strategies (Anderson et al. 2025). Other common active controls were pain education (10 studies) (Patterson et al. 1992; Melzack et al. 1996; Vanhaudenhuyse et al. 2015; Garland et al. 2017; Rizzo et al. 2018; Jensen et al. 2020; Mendoza et al. 2022; Williams et al. 2022; Jensen et al. 2023; Anastas et al. 2024) and CBT (8 studies) (Syrjala et al. 1992; Castel et al. 2009; Castel et al. 2012; Jensen et al. 2020; Bicego et al. 2021; Hanley et al. 2021; Mendoza et al. 2022; Jensen et al. 2023). Pain education involved psychoeducation about pain and modulation (e.g., spinal cord mechanisms, pain ≠ harm) (Knafo and Weinberger 2024) and coping strategies; CBT typically targeted maladaptive appraisals and skills development, including behavioural strategies (Williams et al. 2020). One study blended CBT with pain education (Anderson et al. 2025). Additional active controls not pooled included music (Nilsson et al. 2001; Nilsson et al. 2003; Mackey 2010; Téllez et al. 2016; Mackey 2018; Sánchez‐Jáuregui et al. 2019; Bicego et al. 2021), mindfulness (Garland et al. 2017; Jensen et al. 2018; Hanley et al. 2021; Williams et al. 2022; Anastas et al. 2024), self‐care (Bicego et al. 2021) and stress‐reduction (Frenay et al. 2001). Physiological controls included biofeedback (Jensen et al. 2018), low FODMAP diet (IBS) (Peters et al. 2016), massage (Vanhaudenhuyse et al. 2015; Ardigo et al. 2016), acupuncture (Tastan et al. 2018; Razak et al. 2019) and TENS (Tonye‐Geoffroy et al. 2021). Additionally, as none of the comparisons between combined therapy (i.e., hypnosis combined with an active intervention) and hypnosis or the active intervention only reached the critical mass required for meta‐analysis, the effect sizes were not pooled and were instead narratively reported.

Narrative synthesis of studies not suitable for pooling provided no consistent evidence that hypnosis produced greater pain reduction than other active comparators. Findings were mixed for music and physiological treatments, while hypnosis generally did not differ significantly from mindfulness, stress‐reduction training, self‐care, TENS, acupuncture, massage or low FODMAP diet, with isolated study‐level advantages insufficient to alter the overall conclusion (see Data S5 for full details).

### 3.4 Risk of Bias Assessment

**FIGURE 2:**
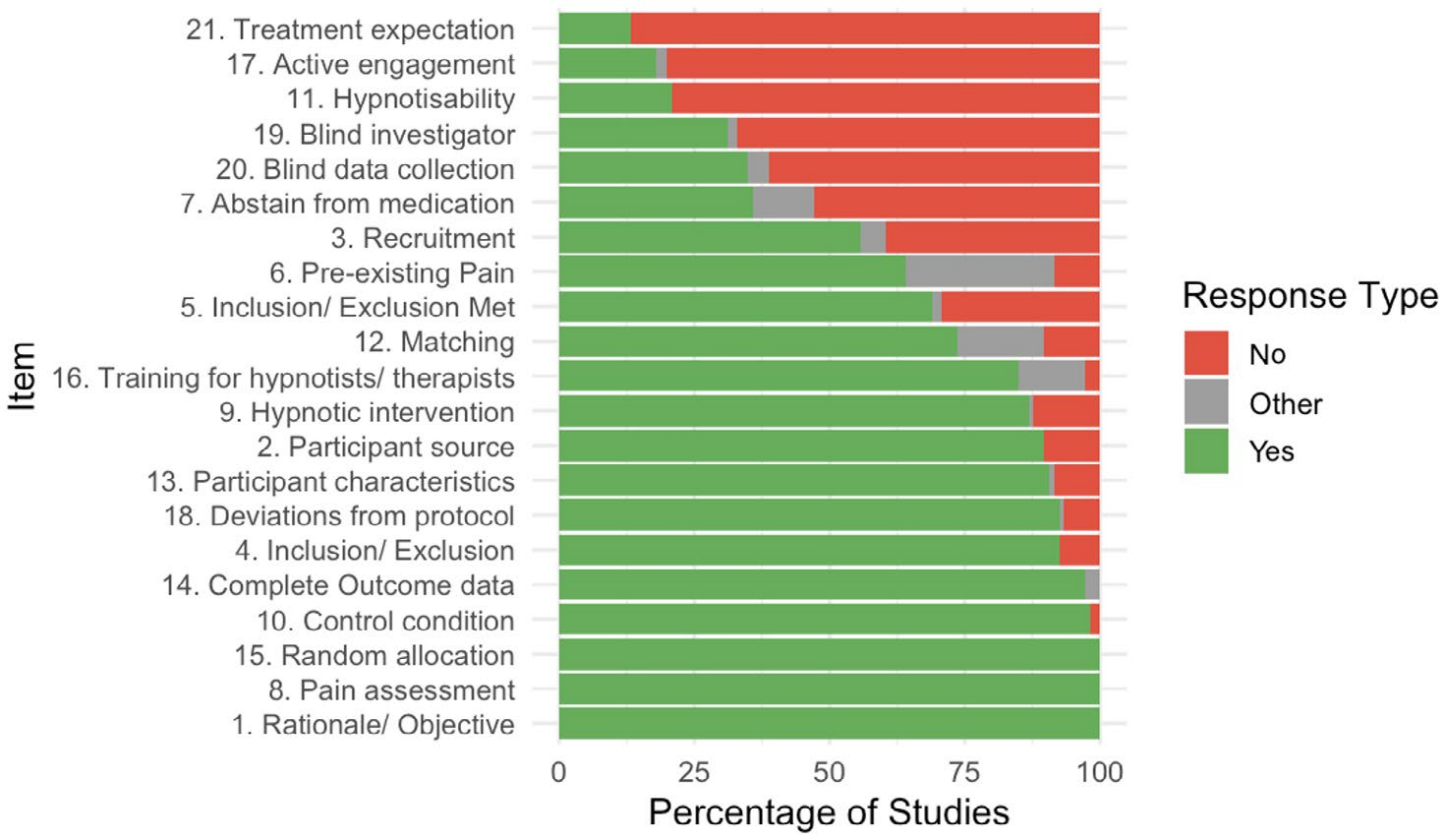
Graph showing the percentage of studies meeting each of the 21 RoB assessment criteria. n.c, sample size of control group; n.e, sample size of experimental group; SE, standard error of standardised mean difference; SMD, standardised mean difference.

Inter‐rater reliability for RoB criteria ranged from κ = −0.02 to 0.85, with weak to almost perfect agreement across 11 domains and perfect agreement in one domain (Data S2). No/minimal agreement (0 ≤ κ ≤ 0.39) occurred in 10 domains (inclusion/exclusion met, pre‐existing pain, abstinence/reporting of analgesic use, hypnotic intervention, matching, complete outcome data, random allocation, training for hypnotist/therapist, active engagement and deviation from protocol) and disagreement (κ < 0) occurred for reporting participant characteristics; ambiguity and inconsistent reporting likely contributed, and discrepancies were resolved by consensus. After resolution, 58 studies (54.72%) were rated low RoB, 44 (41.51%) moderate and 4 (3.77%) high (Data S3). Most notably, many studies did not report treatment expectations (92; 86.8%), active engagement (85; 80.2%), hypnotisability (84; 79.2%), investigator blinding (71; 67.0%), data collection blinding (65; 61.3%) or abstinence from medication (56; 52.8%; Figure 2).

### 3.5 Primary Findings: Hypnosis Versus Non‐Active Controls

Fifty‐eight studies (*n* = 4621; hypnosis *n* = 2351; control *n* = 2270) reported post‐intervention pain intensity comparing hypnosis with non‐active/minimal controls (e.g., distraction, treatment as usual and waitlist). Hypnosis reduced post‐intervention pain versus controls (SMD −0.38; 95% CI −0.56 to −0.21; *t* = −4.37; *p* < 0.0001), with high heterogeneity (*τ* ^2^ = 0.29; 95% CI 0.22–0.63; *I* ^2^ = 76.4%; 95% CI 69.7%–81.6%; *p* < 0.0001). One outlier (Dorta et al. 2024) (SMD −4.03; moderate RoB) contributed substantially.

**FIGURE 3:**
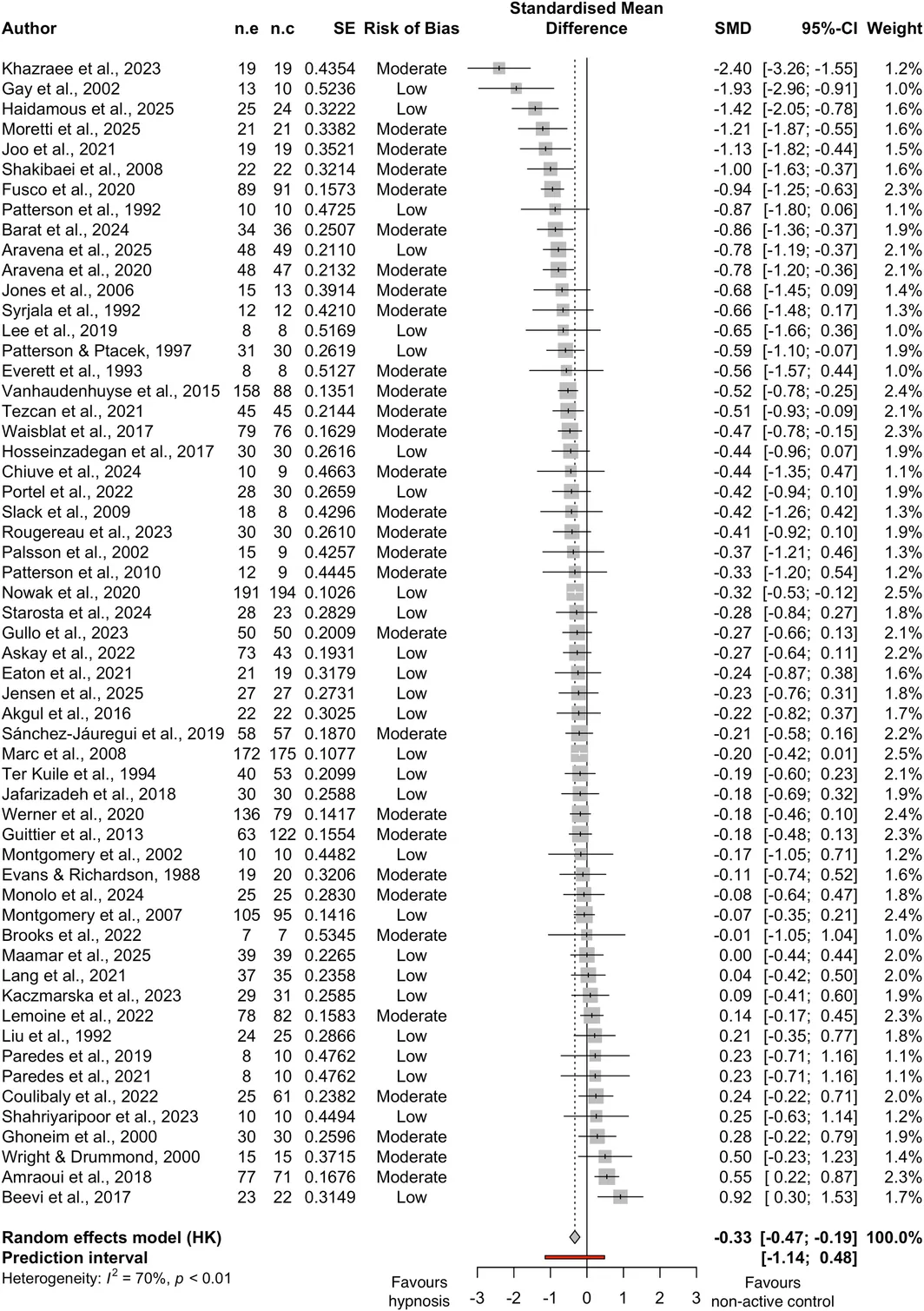
Forest plot comparing effects of hypnosis or non‐active controls on post‐intervention pain intensity (Abbreviations as for Figure 2).

Excluding the outlier yielded 57 studies (*n* = 4572; hypnosis *n* = 2327; control *n* = 2245), with a slightly smaller but still significant effect (SMD −0.33; 95% CI −0.47 to −0.19; *t* = −4.79; *p* < 0.0001; Figure 3) and reduced heterogeneity (*τ* ^2^ = 0.16; 95% CI 0.10–0.34; *I* ^2^ = 70.3%; 95% CI 61.1%–77.2%; *p* < 0.01).

Subgroup analyses showed no significant differences when comparing low (27 studies) with moderate (30 studies) RoB (low: SMD −0.25; 95% CI −0.44 to −0.07; moderate: SMD −0.40; 95% CI −0.61 to −0.20; *p* = 0.26); control type (placebo, 17 studies: SMD −0.31; 95% CI −0.47 to −0.15; waitlist, 9 studies: SMD −0.52; 95% CI −0.83 to −0.21; treatment‐as‐usual, 31 studies: SMD −0.30; 95% CI −0.53 to −0.07; *p* = 0.36); pain type (acute pain, 40 studies: SMD −0.30; 95% CI −0.45 to −0.15; chronic pain, 17 studies: SMD −0.42; 95% CI −0.75 to −0.07; *p* = 0.50); or hypnosis modality (self‐hypnosis, eight studies: SMD −0.24; 95% CI −0.46 to −0.02; therapist‐delivered, 24 studies: SMD −0.35; 95% CI −0.57 to −0.13; in‐person tape, nine studies: SMD −0.33; 95% CI −0.74 to 0.08; therapist‐delivered with self‐hypnosis practice, 10 studies: SMD −0.39; 95% CI −0.99 to 0.22; virtual‐reality, six studies: SMD −0.31; 95% CI −0.75 to 0.13; *p* = 0.94).

**FIGURE 4:**
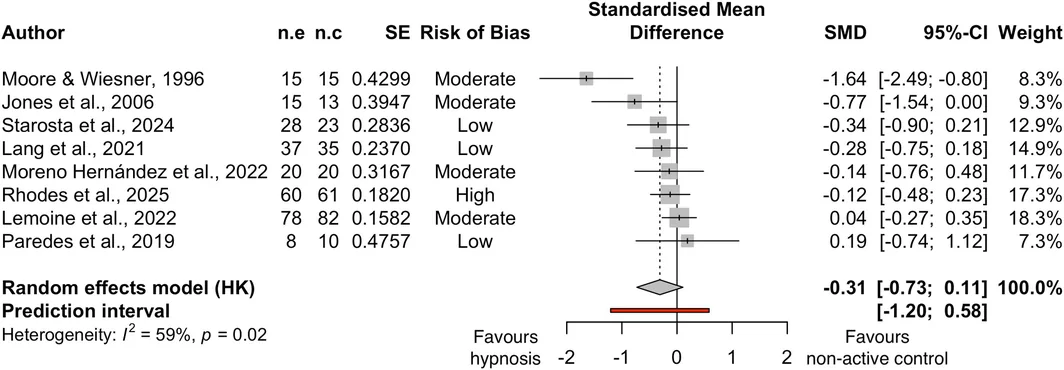
Forest plot comparing the effects of hypnosis or non‐active controls on pain intensity mean difference (Abbreviations as for Figure 2).

Eight studies (*n* = 520; hypnosis *n* = 261; control *n* = 259) reported pre–post change scores (Figure 4). Hypnosis showed a larger decrease than controls, but was not significant (SMD −0.31; 95% CI −0.73 to 0.11; *t* = −1.76; *p* = 0.12), with substantial heterogeneity (*τ* ^2^ = 0.11; 95% CI 0.01–1.24; *I* ^2^ = 58.7%; 95% CI 9.7%–81.1%; *p* = 0.002).

**FIGURE 5:**
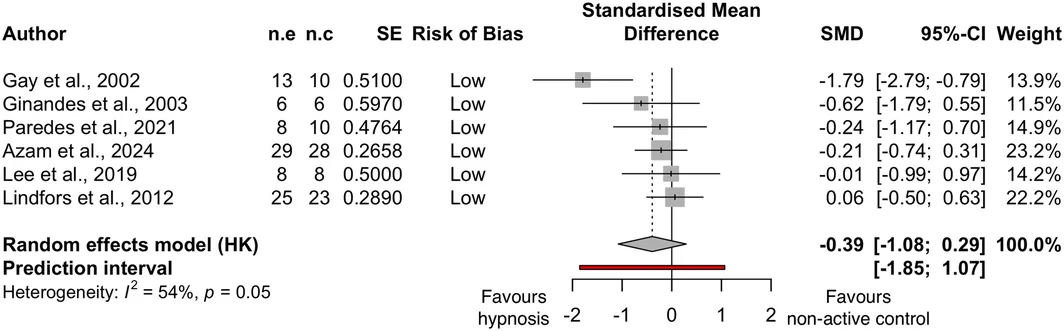
Forest plot comparing effects of hypnosis or non‐active controls on post‐intervention pain intensity at 1–3 months follow‐up (Abbreviations as for Figure 2).

Seven studies (*n* = 223; hypnosis *n* = 113; control *n* = 110) reported post‐intervention pain at 1–3 months follow‐up. The pooled effect was large but non‐significant (SMD −0.96; 95% CI −2.35 to 0.43; *t* = −1.70; *p* = 0.14) with high heterogeneity (*τ* ^2^ = 2.02; 95% CI 0.71–10.84; *I* ^2^ = 90.1%; 95% CI 82.2%–94.5%; *p* < 0.0001), again influenced by outlier (Dorta et al. 2024) (SMD −4.11; moderate RoB). Excluding the outlier (Dorta et al. 2024) left six studies (*n* = 174; hypnosis *n* = 89; control *n* = 85; Figure 5), with a smaller non‐significant effect (SMD −0.39; 95% CI −1.08 to 0.29; *t* = −1.48; *p* = 0.20) and lower heterogeneity (*τ* ^2^ = 0.21; 95% CI 0.00–2.63; *I* ^2^ = 53.9%; 95% CI 0.0%–81.6%; *p* = 0.05).

### 3.6 Primary Findings: Hypnosis Versus Relaxation

**FIGURE 6:**
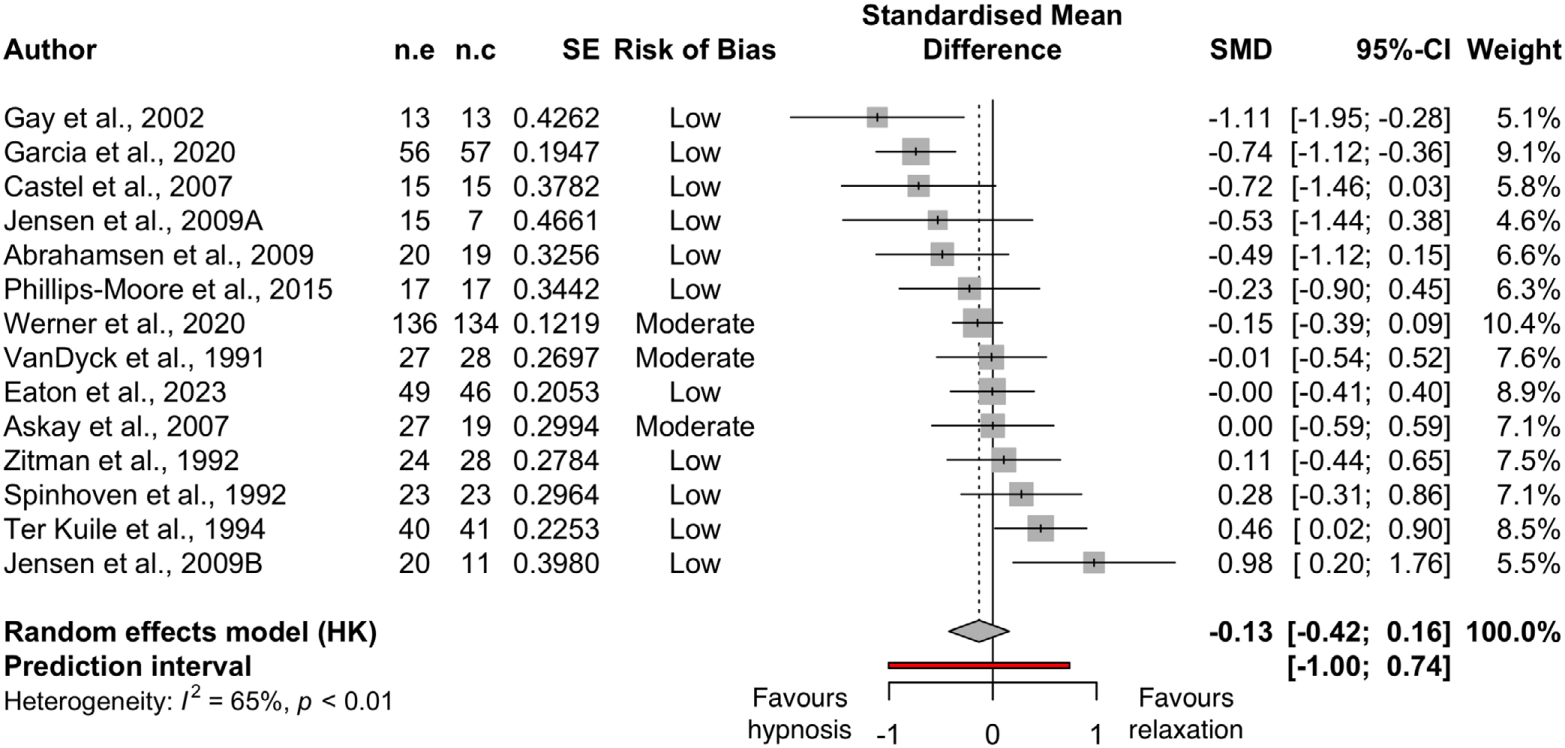
Forest plot comparing the effects of hypnosis or relaxation on post‐intervention pain intensity (Abbreviations as for Figure 2).

Fourteen studies (*n* = 940; hypnosis *n* = 482; relaxation *n* = 458) compared hypnosis with relaxation on post‐intervention pain (Figure 6). The pooled effect was small and non‐significant (SMD −0.13; 95% CI −0.42 to 0.16; *t* = −0.98; *p* = 0.34), with substantial heterogeneity (*τ* ^2^ = 0.14; 95% CI 0.04–0.63; *I* ^2^ = 65.4%; 95% CI 39.0%–80.4%; *p* = 0.0003). Subgroup analyses by RoB showed no significant difference (*p* = 0.64), with low RoB (SMD −0.08) and moderate RoB (SMD −0.31).

### 3.7 Secondary Findings: Hypnosis Versus Pain Education

**FIGURE 7:**
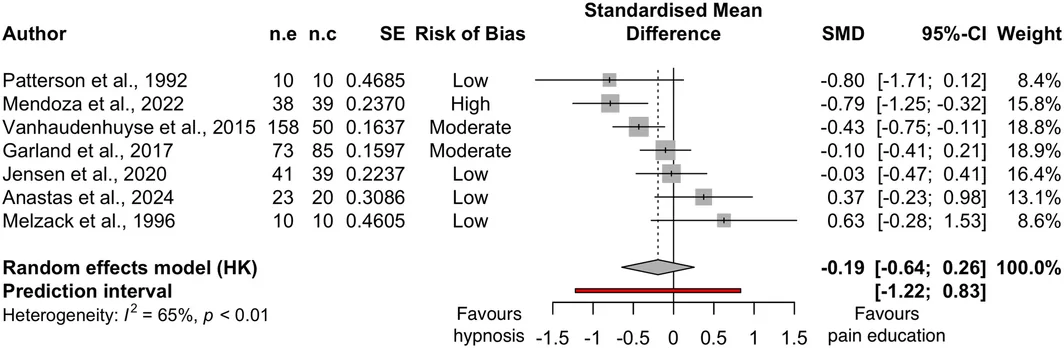
Forest plot comparing the effects of hypnosis or pain education on post‐intervention pain intensity (Abbreviations as for Figure 2).

Seven studies (*n* = 606; hypnosis *n* = 353; pain education *n* = 253) compared hypnosis with pain education on post‐intervention pain (Figure 7). The effect was small and non‐significant (SMD −0.19; 95% CI −0.64 to 0.26; *t* = −1.05; *p* = 0.33), with substantial heterogeneity (*τ* ^2^ = 0.13; 95% CI 0.01–1.30; *I* ^2^ = 65.2%; 95% CI 21.8%–84.5%; *p* = 0.008).

**FIGURE 8:**
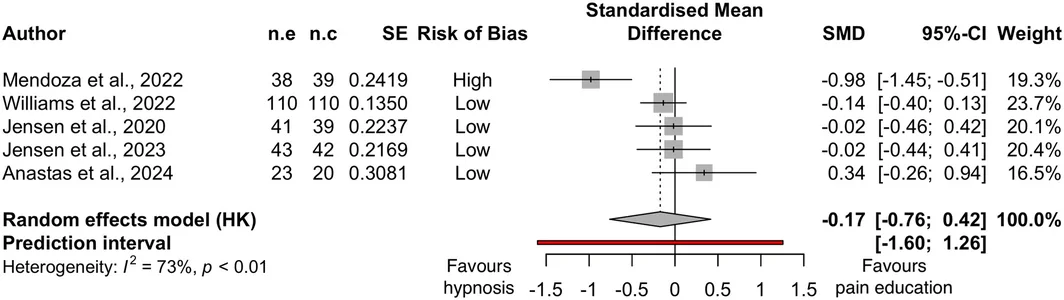
Forest plot comparing the effects of hypnosis or pain education on pain intensity mean difference (Abbreviations as for Figure 2).

Five studies (*n* = 505; hypnosis *n* = 255; pain education *n* = 250) reported pre–post change scores (Figure 8). The pooled effect was small and non‐significant (SMD −0.17; 95% CI −0.76 to 0.42; *t* = −0.81; *p* = 0.46), with substantial heterogeneity (*τ* ^2^ = 0.16; 95% CI 0.02–1.92; *I* ^2^ = 73.3%; 95% CI 33.3%–89.3%; *p* = 0.005).

### 3.8 Secondary Findings: Hypnosis Versus Cognitive Behavioural Therapy

**FIGURE 9:**
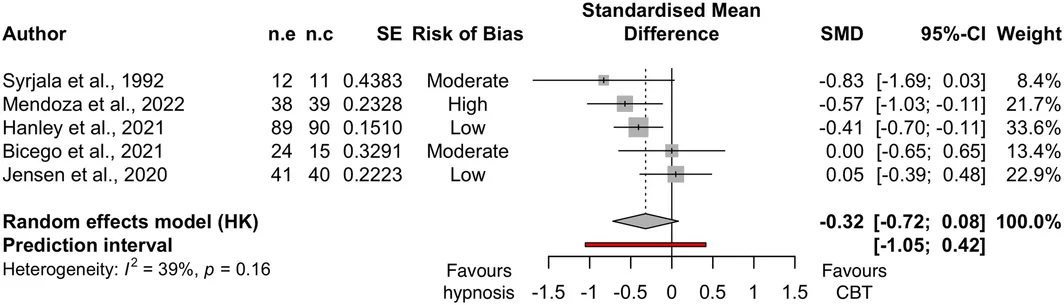
Forest plot comparing the effects of hypnosis or cognitive‐behavioural therapy on post‐intervention pain intensity (Abbreviations as for Figure 2).

Five studies (*n* = 399; hypnosis *n* = 204; CBT *n* = 195) compared hypnosis with CBT on post‐intervention pain (Figure 9). The pooled effect was not statistically significant (SMD −0.32; 95% CI −0.72 to −0.08; *t* = −2.21; *p* = 0.09), with low‐to‐moderate heterogeneity (*τ* ^2^ = 0.03; 95% CI 0.00 –1.04; *I* ^2^ = 38.6%; 95% CI 0.00%–77.2%; *p* = 0.16).

### 3.9 Bias and Sensitivity Analyses

**FIGURE 10:**
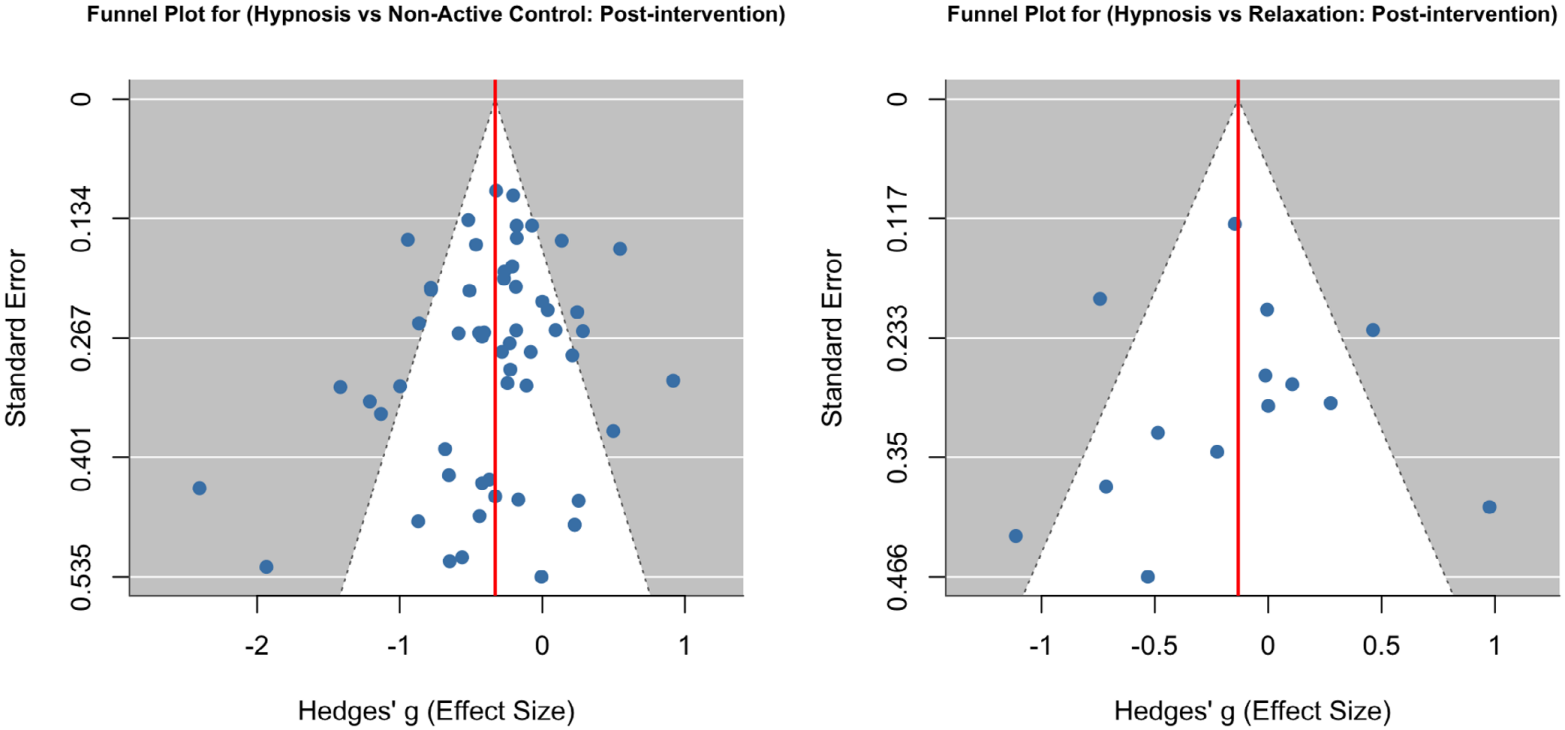
Funnel plots of studies comparing the effects of hypnosis and non‐active control (left) or relaxation (right) on post‐intervention pain intensity.

Excluding high‐RoB studies did not change directions of effect or statistical significance. Visual inspection of funnel plots for primary analyses with > 10 studies suggested no substantial asymmetry (Figure 10). For hypnosis versus non‐active controls (post‐intervention pain), Egger's test was non‐significant (*t* = −1.16, df = 55, *p* = 0.25), indicating no evidence of small‐study effects.

### 3.10 Level of Evidence in Meta‐Analyses

Because included studies were RCTs, the evidence base received an initial ‘high certainty’ rating. Evidence was not downgraded for limitations in study design/execution, indirectness or dissemination bias. Evidence was downgraded for inconsistency (substantial heterogeneity across most meta‐analyses) and imprecision (most comparisons had small‐to‐moderate sample sizes and/or non‐significant effects, except hypnosis versus non‐active controls on post‐intervention pain, *n* = 4572). Final certainty was ‘moderate’ for hypnosis versus non‐active controls and hypnosis versus CBT (post‐intervention pain), and ‘low’ for other meta‐analytic conclusions (Data S4).

## 4 Discussion

To the best of our knowledge, this is the largest meta‐analysis to date critically summarising the analgesic efficacy of hypnosis compared with minimal or non‐active controls (placebos, waitlists and treatment as usual) and active controls (relaxation, CBT and pain education). Across the primary and secondary aims of this systematic review, results converged on a consistent conclusion: hypnosis produces, at best, modest reductions in pain intensity that are comparable to those achieved by other psychological interventions.

### 4.1 Hypnosis Versus Non‐Active Controls or Minimal Intervention Comparisons

Compared to non‐active controls or minimal intervention, aggregate post‐intervention pain intensity following hypnosis was reduced by less than one point on a 1–10 scale. Analyses based on pre–post difference scores, arguably a more conservative indicator of treatment‐related change, yielded a similarly small and non‐significant effect. These modest effects persisted after excluding outlier studies and studies at high risk of bias. Taken together, the observed reductions are unlikely to be experienced by most patients as meaningful improvement, if detected at all (Ferreira et al. 2012; Strijkers et al. 2021).

### 4.2 Hypnosis Versus Active Controls

Beyond comparisons with non‐active controls, hypnosis did not demonstrate superior analgesic effects relative to active interventions, including relaxation, pain education and CBT; effect size differences were generally smaller than those observed against non‐active controls. Narrative synthesis of studies comparing hypnosis with other sensory (e.g., music), psychological (e.g., mindfulness) and physiological interventions (e.g., massage, acupuncture, TENS and biofeedback) likewise provided no basis to revise the central conclusion. Notably, mindfulness and music produced reductions in pain intensity comparable to hypnosis, reinforcing the interpretation that hypnosis does not occupy a privileged position among non‐pharmacological pain treatments.

### 4.3 Factors That Influence Hypnosis Efficacy

Subgroup analyses did not identify reliable factors influencing hypnosis efficacy. More extensive intervention protocols (i.e., therapist‐delivered hypnosis with self‐hypnosis practice) produced the largest reduction in pain intensity (SMD −0.39). Hypnosis was also more effective at reducing chronic pain than acute or procedural pain (acute pain: SMD −0.30 vs. chronic pain: SMD −0.42). Nevertheless, all observed effect sizes were small, and neither more extensive intervention protocols (e.g., therapist‐delivered hypnosis with self‐practice) nor pain type (acute vs. chronic) significantly improved the small effect sizes observed. Future studies or reviews could consider whether the brevity of most hypnosis interventions provides sufficient opportunity to learn or practise hypnotic analgesia, and whether chronic pain patients have greater motivation and more opportunity to practise self‐regulatory strategies. Patient characteristics, pain context and intervention features remain potential moderators of hypnosis response and are worthy of further exploration.

The absence of identifiable moderators in this review may reflect the high heterogeneity of effect sizes across analyses, indicating that hypnosis effects are highly variable. An umbrella review reported wide‐ranging effects of hypnosis on clinical and procedural pain, with effect sizes spanning *d* = 0.37–0.81 and *d* = 0.10–2.53, respectively (Rosendahl et al. 2024). In some circumstances, hypnosis can provide notable pain relief: hypnotic suggestions can effectively modulate experimental pain (Thompson et al. 2019), laboratory studies report substantial control over clinical pain (Derbyshire et al. 2009), and there are reports of childbirth, oral surgery or coronary artery bypass grafting proceeding with minimal pain using hypnoanalgesic procedures alone (Akgul et al. 2016; Venkiteswaran and Tandon 2021; Fernández‐Gamero et al. 2024). Nevertheless, our meta‐analysis provides little evidence for consistent or reliable aggregate pain relief. The high heterogeneity and absence of clear moderators suggest that hypnosis efficacy cannot be predicted from clinical presentation or treatment characteristics, and thus undermine the utility of hypnosis as a scalable, targeted intervention.

### 4.4 Implications of Findings

Overall, pain relief following hypnosis is comparable to that achieved with other psychological therapies, which typically yield small effect sizes (0.1–0.4) corresponding to minimal changes in pain reports (Morley et al. 1999; Veehof et al. 2016; Williams et al. 2020; Strijkers et al. 2021). Our results, alongside other systematic reviews, suggest that any psychological processes exacerbating pain intensity may be difficult to modify with psychological therapy (Veehof et al. 2016; Vlaeyen and Linton 2000; Williams et al. 2020; Strijkers et al. 2021; Turk 2003; Moseley and Butler 2015).

These results align with broader concerns about the limited population‐level impact of many medical interventions despite plausible mechanisms (Stegenga 2018). Examples include antibiotics for acute sinusitis, which only cure one in 17 patients within the recommended treatment period (Lemiengre et al. 2018), statins, which do not prevent death in patients with no known heart disease (Ray et al. 2010), prostate‐specific antigen tests, which do not protect against death from prostate cancer (Djulbegovic et al. 2010), and routine health checks, which do not reduce mortality (Krogsbøll et al. 2019). In this context, modest and inconsistent effects of hypnosis are not anomalous; rather, they reflect a recurring pattern in evidence‐based medicine.

This does not imply that hypnosis should be excluded from clinical care. Unlike medical interventions with meaningful adverse‐effect profiles (e.g., statin‐associated muscle symptoms or myopathy; opioid addiction risk; NSAID‐related gastrointestinal bleeding, renal impairment and cardiovascular risk), psychological interventions are often part of a ‘gentle’ medicine involving less aggressive approaches to ill health (Stegenga 2018). Recommendations to use hypnosis should therefore be grounded in an honest appraisal: it is generally low risk and may help some patients manage pain‐related distress or improve coping even when pain intensity is unchanged (Stegenga 2018). There also remains the possibility that further development of psychological methods arising from better mechanistic understanding and more personalised treatment will yield more successful psychological approaches to pain management.

### 4.5 Study Strengths and Limitations

A key strength of this meta‐analysis is the robustness of results across sensitivity analyses. Removing all studies rated as high risk of bias did not materially alter pooled estimates or change conclusions regarding statistical significance, suggesting that findings were not driven by methodologically weaker studies. In addition, we found no strong evidence of publication bias across primary analyses, reducing concern that the results reflect selective reporting. Together, these features strengthen confidence in the overall pattern of findings.

Several limitations warrant consideration. First, most analyses relied on post‐intervention pain intensity rather than pre–post change scores. This approach implicitly assumes baseline equivalence between groups, an assumption that may not always hold (26.4% of included studies did not report evidence of group matching). Second, pooling self‐reported VAS or NRS measures across studies can be questioned: pain is multidimensional and cannot be captured by intensity alone. Future reviews should evaluate additional outcomes, including pain interference, pain unpleasantness and related functional or affective measures. In addition, while our systematic review does not provide evidence of hypnosis substantially reducing pain intensity, the modest average effect sizes likely obscure a mix of effect sizes for different people and different subgroups. Some patients do experience meaningful pain relief, while others experience other beneficial effects, such as a return to work, elevated mood and a general ability to manage their lives despite their pain. Future investigations might focus less on aggregate pain intensity measures and more on what makes the experience of patients different.

Finally, risk‐of‐bias assessment inevitably involves judgement. The selected criteria and scoring are open to dispute, and disagreements arose both between—and at times within—the current authors. For instance, it is unclear what behavioural observations or procedural requirements are suitable to indicate patient engagement with hypnosis therapy (e.g., pain diaries, engagement questionnaires, qualitative indicators or other indicators of compliance). Similar issues arise in determining what counts as ‘adequate detail’ or ‘clearly reported’, underscoring the subjective elements and interpretation that accompany RoB assessments in complex behavioural interventions. Nevertheless, subgroup analysis based on RoB ratings did not affect the outcomes, and previous systematic reviews while being diligent about assessing RoB still focus on the overall findings without excessive concern about the variable RoB. Reviewers frequently disagree on study quality, leaving comprehensiveness as the most defensible strategy, which this review adopted (Stegenga 2018).

## 5 Conclusion

To the best of our knowledge, this is the largest systematic review and meta‐analysis examining the analgesic effects of hypnosis on clinical pain compared with non‐active/minimal interventions and active controls. Across a wide range of pain conditions, delivery modalities and comparators, hypnosis was associated with small and inconsistent reductions in pain intensity that rarely reached thresholds of clinical importance and did not significantly exceed the effects of other psychological interventions. Future primary studies and systematic reviews should prioritise broader pain outcomes (e.g., pain unpleasantness, pain interference and perceived control) and longer‐term follow‐up to better characterise the role of hypnosis within longer‐term and comprehensive pain management. Overall, these findings support a recalibration of expectations regarding hypnosis for pain intensity reduction: clearer framing of its likely benefits will better serve patients, clinicians and the scientific field.

## Author Contributions

This review was designed by T.H.Y. and S.W.G.D. The searches, assessments and date extraction were completed by T.H.Y. and S.W.G.D. The data were analysed by T.H.Y. and the results interpreted by both authors. S.W.G.D. had a primary role in preparing the manuscript, which was edited by T.H.Y. Both authors have approved the final version of the manuscript and agree to be accountable for all aspects of the work.

## Funding

The authors have nothing to report.

## Conflicts of Interest

The authors declare no conflicts of interest.

## Supporting information


**Data S1:** PRISMA checklist.

**Data S2:** Full RoB Assessment Ratings.

**Data S3:** Full Inter‐rater Reliability for RoB Assessment.

**Data S4:** GRADE Level of Evidence Assessment.

**Data S5:** Narrative synthesis of studies excluded from the meta‐analysis.

## Data Availability

All data are available upon request to the corresponding author.

## References

[ejp70369-bib-0001] Abrahamsen, R. , L. Baad‐Hansen , and P. Svensson . 2008. “Hypnosis in the Management of Persistent Idiopathic Orofacial Pain—Clinical and Psychosocial Findings.” Pain 136: 44–52. 10.1016/j.pain.2007.06.013. 17689192

[ejp70369-bib-0002] Abrahamsen, R. , R. Zachariae , and P. Svensson . 2009. “Effect of Hypnosis on Oral Function and Psychological Factors in Temporomandibular Disorders Patients.” Journal of Oral Rehabilitation 36: 556–570. 10.1111/j.1365-2842.2009.01974.x. 19604319

[ejp70369-bib-0003] Adachi, T. , H. Fujino , A. Nakae , T. Mashimo , and J. Sasaki . 2014. “A Meta‐Analysis of Hypnosis for Chronic Pain Problems: A Comparison Between Hypnosis, Standard Care, and Other Psychological Interventions.” International Journal of Clinical and Experimental Hypnosis 62: 1–28. 10.1080/00207144.2013.841471. 24256477

[ejp70369-bib-0004] Akgul, A. , B. Guner , M. Çırak , D. Çelik , O. Hergünsel , and S. Bedirhan . 2016. “The Beneficial Effect of Hypnosis in Elective Cardiac Surgery: A Preliminary Study.” Thoracic and Cardiovascular Surgeon 64: 581–588. 10.1055/s-0036-1580623. 27043785

[ejp70369-bib-0005] Amraoui, J. , C. Pouliquen , J. Fraisse , et al. 2018. “Effects of a Hypnosis Session Before General Anesthesia on Postoperative Outcomes in Patients Who Underwent Minor Breast Cancer Surgery: The HYPNOSEIN Randomized Clinical Trial.” JAMA Network Open 1: e181164. 10.1001/jamanetworkopen.2018.1164. 30646110 PMC6324272

[ejp70369-bib-0006] Anastas, T. M. , A. P. Turner , E. J. Ho , et al. 2024. “Evaluating the Benefits of a Second Pain Treatment Following a Clinical Trial.” Rehabilitation Psychology 69: 74–83. 10.1037/rep0000510. 37338442 PMC10728346

[ejp70369-bib-0007] Anderson, E. J. , S. L. Peters , P. R. Gibson , and E. P. Halmos . 2025. “Comparison of Digitally Delivered Gut‐Directed Hypnotherapy Program With an Active Control for Irritable Bowel Syndrome.” American Journal of Gastroenterology 120: 440–448. 10.14309/ajg.0000000000002921. 38940439

[ejp70369-bib-0009] Aravena, V. , F. E. García , A. Téllez , and P. R. Arias . 2020. “Hypnotic Intervention in People With Fibromyalgia: A Randomized Controlled Trial.” American Journal of Clinical Hypnosis 63: 49–61. 10.1080/00029157.2020.1742088. 32744483

[ejp70369-bib-0008] Aravena, V. , F. García , A. Téllez , and P. Arias . 2025. “Hypnosis, Pain, Affectivity and Quality of Life in Patients With Fibromyalgia: A Randomized Controlled Trial.” Terapia Psicológica 43: 203–222. 10.4067/s0718-48082025000200203.

[ejp70369-bib-0010] Ardigo, S. , F. R. Herrmann , V. Moret , et al. 2016. “Hypnosis Can Reduce Pain in Hospitalized Older Patients: A Randomized Controlled Study.” BMC Geriatrics 16: 14. 10.1186/s12877-016-0180-y. 26767506 PMC4714456

[ejp70369-bib-0011] Askay, S. W. , M. P. Jensen , S. R. Sharar , J. K. Barber , M. Soltani , and D. R. Patterson . 2022. “The Impact of Virtual Reality Hypnosis on Pain and Anxiety Caused by Trauma: Lessons Learned From a Clinical Trial.” International Journal of Clinical and Experimental Hypnosis 70: 156–173. 10.1080/00207144.2022.2052296. 35348435 PMC9248347

[ejp70369-bib-0012] Askay, S. W. , D. Patterson , M. Jensen , and S. Sharar . 2007. “A Randomized Controlled Trial of Hypnosis for Burn Wound Care.” Rehabilitation Psychology 52: 247–253. 10.1037/0090-5550.52.3.247.

[ejp70369-bib-0013] Azam, M. A. , A. Z. Weinrib , P. M. Slepian , et al. 2024. “Effects of Perioperative Clinical Hypnosis on Heart Rate Variability in Patients Undergoing Oncologic Surgery: Secondary Outcomes of a Randomized Controlled Trial.” Frontiers in Pain Research (Lausanne) 5: 1354015. 10.3389/fpain.2024.1354015. PMC10957530 38524266

[ejp70369-bib-0014] Barat, M. , C. Ollivier , L. Taibi , et al. 2025. “Standard of Care Versus Standard of Care Plus Ericksonian Hypnosis for Percutaneous Liver Biopsy: Results of a Randomized Control Trial.” Diagnostic and Interventional Imaging 106: 93–97. 10.1016/j.diii.2024.09.009. 39358154

[ejp70369-bib-0015] Barber, J. 1977. “Rapid Induction Analgesia: A Clinical Report.” American Journal of Clinical Hypnosis 19: 138–145. 10.1080/00029157.1977.10403860. 835486

[ejp70369-bib-0016] Bartley, E. J. , and R. B. Fillingim . 2013. “Sex Differences in Pain: A Brief Review of Clinical and Experimental Findings.” British Journal of Anaesthesia 111: 52–58. 10.1093/bja/aet127. 23794645 PMC3690315

[ejp70369-bib-0017] Beevi, Z. , W. Y. Low , and H. Jamiyah . 2017. “The Effectiveness of Hypnosis Intervention for Labor: An Experimental Study.” American Journal of Clinical Hypnosis 60: 172–191. 10.1080/00029157.2017.1280659. 28891771

[ejp70369-bib-0018] Bicego, A. , J. Monseur , A. Collinet , et al. 2021. “Complementary Treatment Comparison for Chronic Pain Management: A Randomized Longitudinal Study.” PLoS One 16: e0256001. 10.1371/journal.pone.0256001. 34358272 PMC8345881

[ejp70369-bib-0019] Brooks, T. , R. Sharp , S. Evans , S. Scharfbillig , J. Baranoff , and A. Esterman . 2022. “Potential Feasibility of an Online Hypnosis Intervention for Women With Persistent Pelvic Pain.” International Journal of Clinical and Experimental Hypnosis 70: 196–207. 10.1080/00207144.2022.2052297. 35344474

[ejp70369-bib-0020] Castel, A. , R. Cascón , A. Padrol , J. Sala , and M. Rull . 2012. “Multicomponent Cognitive‐Behavioral Group Therapy With Hypnosis for the Treatment of Fibromyalgia: Long‐Term Outcome.” Journal of Pain 13: 255–265. 10.1016/j.jpain.2011.11.005. 22285609

[ejp70369-bib-0021] Castel, A. , M. Pérez , J. Sala , A. Padrol , and M. Rull . 2007. “Effect of Hypnotic Suggestion on Fibromyalgic Pain: Comparison Between Hypnosis and Relaxation.” European Journal of Pain 11: 463–468. 10.1016/j.ejpain.2006.06.006. 16889999

[ejp70369-bib-0022] Castel, A. , M. Salvat , J. Sala , and M. Rull . 2009. “Cognitive‐Behavioural Group Treatment With Hypnosis: A Randomized Pilot Trial in Fibromyalgia.” Contemporary Hypnosis 26: 48–59. 10.1002/ch.372.

[ejp70369-bib-0023] Chiuvé, S. C. , S. Momjian , A. Wolff , and M. V. Corniola . 2024. “Effectiveness and Reliability of Hypnosis in Stereotaxy: A Randomized Study.” Acta Neurochirurgica (Wien) 166: 112. 10.1007/s00701-024-05943-0. PMC10899299 38411747

[ejp70369-bib-0024] Cohen, S. P. , L. Vase , and W. M. Hooten . 2021. “Chronic Pain: An Update on Burden, Best Practices, and New Advances.” Lancet 397: 2082–2097. 10.1016/s0140-6736(21)00393-7. 34062143

[ejp70369-bib-0025] Coulibaly, I. , L. S. Cardelli , C. Duflos , et al. 2022. “Virtual Reality Hypnosis in the Electrophysiology Lab: When Human Treatments Are Better Than Virtual Ones.” Journal of Clinical Medicine 11: 3913. 10.3390/jcm11133913. 35807198 PMC9267480

[ejp70369-bib-0026] Derbyshire, S. W. G. , M. G. Whalley , and D. A. Oakley . 2009. “Fibromyalgia Pain and Its Modulation by Hypnotic and Non‐Hypnotic Suggestion: An fMRI Analysis.” European Journal of Pain 13: 542–550. 10.1016/j.ejpain.2008.06.010. 18653363

[ejp70369-bib-0027] Djulbegovic, M. , R. J. Beyth , M. M. Neuberger , et al. 2010. “Screening for Prostate Cancer: Systematic Review and Meta‐Analysis of Randomised Controlled Trials.” BMJ 341: c4543. 10.1136/bmj.c4543. 20843937 PMC2939952

[ejp70369-bib-0028] Dorta, D. C. , P. O. Colavolpe , P. S. S. Lauria , R. B. Fonseca , V. Brito , and C. F. Villarreal . 2024. “Multimodal Benefits of Hypnosis on Pain, Mental Health, Sleep, and Quality of Life in Patients With Chronic Pain Related to Fibromyalgia: A Randomized, Controlled, Blindly‐Evaluated Trial.” Explorer 20: 103016. 10.1016/j.explore.2024.103016. 38879420

[ejp70369-bib-0029] Eaton, L. H. , S. L. Beck , and M. P. Jensen . 2021. “An Audio‐Recorded Hypnosis Intervention for Chronic Pain Management in Cancer Survivors: A Randomized Controlled Pilot Study.” International Journal of Clinical and Experimental Hypnosis 69: 422–440. 10.1080/00207144.2021.1951119. 34309480 PMC8458244

[ejp70369-bib-0030] Eaton, L. H. , M. K. Jang , M. P. Jensen , K. C. Pike , M. M. Heitkemper , and A. Z. Doorenbos . 2023. “Hypnosis and Relaxation Interventions for Chronic Pain Management in Cancer Survivors: A Randomized Controlled Trial.” Support Care Cancer 31: 50. 10.1007/s00520-022-07498-1. 36526937

[ejp70369-bib-0031] Erickson, M. H. , and E. L. Rossi . 1979. Hypnotherapy—An Exploratory Casebook. Irvington.

[ejp70369-bib-0032] Evans, C. , and P. H. Richardson . 1988. “Improved Recovery and Reduced Postoperative Stay After Therapeutic Suggestions During General Anaesthesia.” Lancet 2: 491–493. 10.1016/s0140-6736(88)90131-6. 2900410 PMC3023142

[ejp70369-bib-0033] Everett, J. J. , D. R. Patterson , G. L. Burns , B. Montgomery , and D. Heimbach . 1993. “Adjunctive Interventions for Burn Pain Control: Comparison of Hypnosis and Ativan: The 1993 Clinical Research Award.” Journal of Burn Care & Rehabilitation 14: 676–683. 10.1097/00004630-199311000-00014. 7507933

[ejp70369-bib-0034] Fernández‐Gamero, L. , A. Reinoso‐Cobo , M. D. C. Ruiz‐González , et al. 2024. “Impact of Hypnotherapy on Fear, Pain, and the Birth Experience: A Systematic Review.” Healthcare (Basel) 12: 616. 10.3390/healthcare12060616. 38540580 PMC10970289

[ejp70369-bib-0035] Ferreira, M. L. , R. D. Herbert , P. H. Ferreira , et al. 2012. “A Critical Review of Methods Used to Determine the Smallest Worthwhile Effect of Interventions for Low Back Pain.” Journal of Clinical Epidemiology 65: 253–261. 10.1016/j.jclinepi.2011.06.018. 22014888

[ejp70369-bib-0036] Frenay, M. C. , M. E. Faymonville , S. Devlieger , A. Albert , and A. Vanderkelen . 2001. “Psychological Approaches During Dressing Changes of Burned Patients: A Prospective Randomised Study Comparing Hypnosis Against Stress Reducing Strategy.” Burns 27: 793–799. 10.1016/s0305-4179(01)00035-3. 11718981

[ejp70369-bib-0037] Fusco, N. , F. Bernard , F. Roelants , et al. 2020. “Hypnosis and Communication Reduce Pain and Anxiety in Peripheral Intravenous Cannulation: Effect of Language and Confusion on Pain During Peripheral Intravenous Catheterization (KTHYPE), a Multicentre Randomised Trial.” British Journal of Anaesthesia 124: 292–298. 10.1016/j.bja.2019.11.020. 31862159

[ejp70369-bib-0038] Garcia, R. , C. Bouleti , A. Li , et al. 2020. “Hypnosis Versus Placebo During Atrial Flutter Ablation: The PAINLESS Study: A Randomized Controlled Trial.” JACC Clinical Electrophysiology 6: 1551–1560. 10.1016/j.jacep.2020.05.028. 33213815

[ejp70369-bib-0039] Garland, E. L. , A. K. Baker , P. Larsen , et al. 2017. “Randomized Controlled Trial of Brief Mindfulness Training and Hypnotic Suggestion for Acute Pain Relief in the Hospital Setting.” Journal of General Internal Medicine 32: 1106–1113. 10.1007/s11606-017-4116-9. 28702870 PMC5602767

[ejp70369-bib-0040] Gay, M. C. , P. Philippot , and O. Luminet . 2002. “Differential Effectiveness of Psychological Interventions for Reducing Osteoarthritis Pain: A Comparison of Erikson [Correction of Erickson] Hypnosis and Jacobson Relaxation.” European Journal of Pain 6: 1–16. 10.1053/eujp.2001.0263. 11888223

[ejp70369-bib-0041] GBD 2016 Disease and Injury Incidence and Prevalence Collaborators . 2017. “Global, Regional, and National Incidence, Prevalence, and Years Lived With Disability for 328 Diseases and Injuries for 195 Countries, 1990–2016: A Systematic Analysis for the Global Burden of Disease Study 2016.” Lancet 390: 1211–1259. 10.1016/s0140-6736(17)32154-2. 28919117 PMC5605509

[ejp70369-bib-0042] Ghoneim, M. M. , R. I. Block , D. S. Sarasin , C. S. Davis , and J. N. Marchman . 2000. “Tape‐Recorded Hypnosis Instructions as Adjuvant in the Care of Patients Scheduled for Third Molar Surgery.” Anesthesia and Analgesia 90: 64–68. 10.1097/00000539-200001000-00016. 10624980

[ejp70369-bib-0043] Ginandes, C. , P. Brooks , W. Sando , C. Jones , and J. Aker . 2003. “Can Medical Hypnosis Accelerate Post‐Surgical Wound Healing? Results of a Clinical Trial.” American Journal of Clinical Hypnosis 45: 333–351. 10.1080/00029157.2003.10403546. 12722936

[ejp70369-bib-0044] Guittier, M.‐J. , F. Guillemin , E. Brandao‐Farinelli , O. Irion , M. Boulvain , and B. Martinez de Tejada . 2013. “Hypnosis for the Control of Pain Associated With External Cephalic Version: A Comparative Study.” Journal of Alternative and Complementary Medicine (New York, N.Y.) 19: 820–825. 10.1089/acm.2012.0945. 23461523

[ejp70369-bib-0045] Gullo, G. , D. C. Rotzinger , A. Colin , et al. 2023. “Virtually Augmented Self‐Hypnosis in Peripheral Vascular Intervention: A Randomized Controlled Trial.” Cardiovascular and Interventional Radiology 46: 786–793. 10.1007/s00270-023-03394-1. 36944851 PMC10030078

[ejp70369-bib-0046] Haidamous, G. , M. Jensen , L. Couchara , K. Christmas , P. Simon , and M. Frankle . 2025. “Effects of Hypnosis Therapy on Pain and Opioid Use Following Shoulder Replacement Surgery: A Pilot Feasibility Study.” International Journal of Clinical and Experimental Hypnosis 74: 1–20. 10.1080/00207144.2025.2544059. 40982709

[ejp70369-bib-0047] Hanley, A. W. , J. Gililland , J. Erickson , et al. 2021. “Brief Preoperative Mind‐Body Therapies for Total Joint Arthroplasty Patients: A Randomized Controlled Trial.” Pain 162: 1749–1757. 10.1097/j.pain.0000000000002195. 33449510 PMC8119303

[ejp70369-bib-0048] Hedges, L. , and I. Olkin . 1984. “Nonparametric Estimators of Effect Size in Meta‐Analysis.” Psychological Bulletin 96: 573–580. 10.1037/0033-2909.96.3.573.

[ejp70369-bib-0049] Hosseinzadegan, F. , M. Radfar , A. R. Shafiee‐Kandjani , and N. Sheikh . 2017. “Efficacy of Self‐ Hypnosis in Pain Management in Female Patients With Multiple Sclerosis.” International Journal of Clinical and Experimental Hypnosis 65: 86–97. 10.1080/00207144.2017.1246878. 27935465

[ejp70369-bib-0050] IntHout, J. , J. P. Ioannidis , and G. F. Borm . 2014. “The Hartung‐Knapp‐Sidik‐Jonkman Method for Random Effects Meta‐Analysis Is Straightforward and Considerably Outperforms the Standard DerSimonian‐Laird Method.” BMC Medical Research Methodology 14: 25. 10.1186/1471-2288-14-25. 24548571 PMC4015721

[ejp70369-bib-0051] Jackson, T. , S. Thomas , V. Stabile , M. Shotwell , X. Han , and K. McQueen . 2016. “A Systematic Review and Meta‐Analysis of the Global Burden of Chronic Pain Without Clear Etiology in Low‐ and Middle‐Income Countries: Trends in Heterogeneous Data and a Proposal for New Assessment Methods.” Anesthesia and Analgesia 123: 739–748. 10.1213/ane.0000000000001389. 27537761

[ejp70369-bib-0052] Jafarizadeh, H. , M. Lotfi , F. Ajoudani , A. Kiani , and V. Alinejad . 2018. “Hypnosis for Reduction of Background Pain and Pain Anxiety in Men With Burns: A Blinded, Randomised, Placebo‐Controlled Study.” Burns 44: 108–117. 10.1016/j.burns.2017.06.001. 28801149

[ejp70369-bib-0053] Jenkins, H. J. , L. Corrêa , B. T. Brown , et al. 2025. “Long‐Term Effectiveness of Non‐Surgical Interventions for Chronic Low Back Pain: A Systematic Review and Meta‐Analysis.” Lancet Rheumatology 7: e607–e617. 10.1016/s2665-9913(25)00064-5. 40449512

[ejp70369-bib-0055] Jensen, M. P. , J. Barber , J. M. Romano , et al. 2009a. “Effects of Self‐Hypnosis Training and EMG Biofeedback Relaxation Training on Chronic Pain in Persons With Spinal‐Cord Injury.” International Journal of Clinical and Experimental Hypnosis 57: 239–268. 10.1080/00207140902881007. 19459087 PMC2730649

[ejp70369-bib-0056] Jensen, M. P. , J. Barber , J. M. Romano , et al. 2009b. “A Comparison of Self‐Hypnosis Versus Progressive Muscle Relaxation in Patients With Multiple Sclerosis and Chronic Pain.” International Journal of Clinical and Experimental Hypnosis 57: 198–221. 10.1080/00207140802665476. 19234967 PMC2758639

[ejp70369-bib-0057] Jensen, M. P. , S. L. Battalio , J. F. Chan , et al. 2018. “Use of Neurofeedback and Mindfulness to Enhance Response to Hypnosis Treatment in Individuals With Multiple Sclerosis: Results From a Pilot Randomized Clinical Trial.” International Journal of Clinical and Experimental Hypnosis 66: 231–264. 10.1080/00207144.2018.1460546. 29856281

[ejp70369-bib-0054] Jensen, M. , J. Chan , O. Gottlieb , L. Sugarman , and E. Stensland . 2025. “Use and Benefits of a Digital Therapeutic for Chronic Pain Management: A Pilot Study.” Psychology of Consciousness: Theory, Research and Practice 12: 727–744. 10.1037/cns0000425.

[ejp70369-bib-0058] Jensen, M. P. , D. M. Ehde , S. Hakimian , M. W. Pettet , M. A. Day , and M. A. Ciol . 2023. “Who Benefits the Most From Different Psychological Chronic Pain Treatments? An Exploratory Analysis of Treatment Moderators.” Journal of Pain 24: 2024–2039. 10.1016/j.jpain.2023.06.011. 37353183 PMC10615716

[ejp70369-bib-0059] Jensen, M. P. , M. E. Mendoza , D. M. Ehde , et al. 2020. “Effects of Hypnosis, Cognitive Therapy, Hypnotic Cognitive Therapy, and Pain Education in Adults With Chronic Pain: A Randomized Clinical Trial.” Pain 161: 2284–2298. 10.1097/j.pain.0000000000001943. 32483058 PMC7508809

[ejp70369-bib-0060] Jones, H. , P. Cooper , V. Miller , N. Brooks , and P. J. Whorwell . 2006. “Treatment of Non‐Cardiac Chest Pain: A Controlled Trial of Hypnotherapy.” Gut 55: 1403–1408. 10.1136/gut.2005.086694. 16627548 PMC1856426

[ejp70369-bib-0061] Jones, H. G. , R. R. N. Rizzo , B. W. Pulling , et al. 2024. “Adjunctive Use of Hypnosis for Clinical Pain: A Systematic Review and Meta‐Analysis.” Pain Reports 9: e1185. 10.1097/pr9.0000000000001185. 39263007 PMC11390056

[ejp70369-bib-0062] Joo, Y. , E. K. Kim , H. G. Song , H. Jung , H. Park , and J. Y. Moon . 2021. “Effectiveness of Virtual Reality Immersion on Procedure‐Related Pain and Anxiety in Outpatient Pain Clinic: An Exploratory Randomized Controlled Trial.” Korean Journal of Pain 34: 304–314. 10.3344/kjp.2021.34.3.304. 34193636 PMC8255151

[ejp70369-bib-0154] Kaczmarska, A. D. , M. Mielimąka , and K. Rutkowski . 2023. “The Efficacy of Hypnotic Analgesic Suggestions in Chronic Nociplastic Pain: A Randomized Controlled Trial.” International Journal of Clinical and Experimental Hypnosis 71, no. 3: 216–234. 10.1080/00207144.2023.2226169. 37358865

[ejp70369-bib-0063] Kaczmarska, A. D. , K. Rutkowski , and M. Mielimąka . 2024. “Immediate Hypnosis Effects and Outcome Predictors in Chronic Nociplastic Pain.” American Journal of Clinical Hypnosis 66: 231–242. 10.1080/00029157.2023.2243618. 37707531

[ejp70369-bib-0064] Khazraee, H. , M. Bakhtiari , A. S. Kianimoghadam , and R. Hajmanouchehri . 2023. “The Effectiveness of Mindful Hypnotherapy on Psychological Inflexibility, Pain Acceptance, Headache Disability and Intensity in Females With Chronic Migraine Headache: A Randomized Clinical Trial.” Life (Basel) 13: 131. 10.3390/life13010131. 36676080 PMC9865410

[ejp70369-bib-0065] Knafo, G. , and J. Weinberger . 2024. “Exploring the Role of Conscious and Unconscious Processes in Hypnosis: A Theoretical Review.” Brain Sciences 14: 374. 10.3390/brainsci14040374. 38672023 PMC11048517

[ejp70369-bib-0066] Knapp, G. , and J. Hartung . 2003. “Improved Tests for a Random Effects Meta‐Regression With a Single Covariate.” Statistics in Medicine 22: 2693–2710. 10.1002/sim.1482. 12939780

[ejp70369-bib-0067] Krogsbøll, L. T. , K. J. Jørgensen , and P. C. Gøtzsche . 2019. “General Health Checks in Adults for Reducing Morbidity and Mortality From Disease.” Cochrane Database of Systematic Reviews 1: Cd009009. 10.1002/14651858.CD009009.pub3. 30699470 PMC6353639

[ejp70369-bib-0068] Lang, E. V. , W. Jackson , P. Senn , et al. 2021. “Efficacy of a Self‐Hypnotic Relaxation App on Pain and Anxiety in a Randomized Clinical Trial: Results and Considerations on the Design of Active and Control Apps.” International Journal of Clinical and Experimental Hypnosis 69: 277–295. 10.1080/00207144.2021.1883988. 33724898 PMC9976960

[ejp70369-bib-0069] Langan, D. , J. P. T. Higgins , D. Jackson , et al. 2019. “A Comparison of Heterogeneity Variance Estimators in Simulated Random‐Effects Meta‐Analyses.” Research Synthesis Methods 10: 83–98. 10.1002/jrsm.1316. 30067315

[ejp70369-bib-0070] Langlois, P. , A. Perrochon , R. David , et al. 2022. “Hypnosis to Manage Musculoskeletal and Neuropathic Chronic Pain: A Systematic Review and Meta‐Analysis.” Neuroscience and Biobehavioral Reviews 135: 104591. 10.1016/j.neubiorev.2022.104591. 35192910

[ejp70369-bib-0071] Lau, J. , J. P. Ioannidis , N. Terrin , C. H. Schmid , and I. Olkin . 2006. “The Case of the Misleading Funnel Plot.” BMJ 333: 597–600. 10.1136/bmj.333.7568.597. 16974018 PMC1570006

[ejp70369-bib-0072] Lee, J. K. , J. O. Zubaidah , I. S. I. Fadhilah , I. Normala , and M. P. Jensen . 2019. “Prerecorded Hypnotic Peri‐Surgical Intervention to Alleviate Risk of Chronic Postsurgical Pain in Total Knee Replacement: A Randomized Controlled Pilot Study.” International Journal of Clinical and Experimental Hypnosis 67: 217–245. 10.1080/00207144.2019.1580975. 30939085

[ejp70369-bib-0073] Lemiengre, M. B. , M. L. van Driel , D. Merenstein , H. Liira , M. Mäkelä , and A. I. De Sutter . 2018. “Antibiotics for Acute Rhinosinusitis in Adults.” Cochrane Database of Systematic Reviews 9: Cd006089. 10.1002/14651858.CD006089.pub5. 30198548 PMC6513448

[ejp70369-bib-0074] Lemoine, L. , V. Adam , X. Galus , et al. 2022. “Conversational Hypnosis Versus Standard of Care to Reduce Anxiety in Patients Undergoing Marker Placement Under Radiographic Control Prior to Breast Cancer Surgery: A Randomized, Multicenter Trial.” Frontiers in Psychology 13: 971232. 10.3389/fpsyg.2022.971232. 36483698 PMC9724617

[ejp70369-bib-0075] Lindfors, P. , P. Unge , P. Arvidsson , et al. 2012. “Effects of Gut‐Directed Hypnotherapy on IBS in Different Clinical Settings‐Results From Two Randomized, Controlled Trials.” American Journal of Gastroenterology 107: 276–285. 10.1038/ajg.2011.340. 21971535

[ejp70369-bib-0076] Liu, W. H. , P. J. Standen , and A. R. Aitkenhead . 1992. “Therapeutic Suggestions During General Anaesthesia in Patients Undergoing Hysterectomy.” British Journal of Anaesthesia 68: 277–281. 10.1093/bja/68.3.277. 1547052

[ejp70369-bib-0077] Maamar, A. , C. Faleur , K. Pedrono , et al. 2025. “Hypnosis for Unplanned Procedural Pain in the Intensive Care Unit: The HYPIC Randomized Clinical Trial.” Critical Care 29: 312. 10.1186/s13054-025-05563-9. 40682163 PMC12275327

[ejp70369-bib-0078] Mackey, E. F. 2010. “Effects of Hypnosis as an Adjunct to Intravenous Sedation for Third Molar Extraction: A Randomized, Blind, Controlled Study.” International Journal of Clinical and Experimental Hypnosis 58: 21–38. 10.1080/00207140903310782. 20183736

[ejp70369-bib-0079] Mackey, E. F. 2018. “An Extension Study Using Hypnotic Suggestion as an Adjunct to Intravenous Sedation.” American Journal of Clinical Hypnosis 60: 378–385. 10.1080/00029157.2017.1416279. 29485375

[ejp70369-bib-0080] Marc, I. , P. Rainville , B. Masse , et al. 2008. “Hypnotic Analgesia Intervention During First‐Trimester Pregnancy Termination: An Open Randomized Trial.” American Journal of Obstetrics and Gynecology 199: 469. 10.1016/j.ajog.2008.01.058. 18377854

[ejp70369-bib-0081] McHugh, M. L. 2012. “Interrater Reliability: The Kappa Statistic.” Biochemia Medica (Zagreb) 22: 276–282. PMC3900052 23092060

[ejp70369-bib-0082] Melzack, R. , M. Germain , E. Belanger , P. N. Fuchs , and R. Swick . 1996. “Positive Intrasurgical Suggestion Fails to Affect Postsurgical Pain.” Journal of Pain and Symptom Management 11: 103–107. 10.1016/0885-3924(95)00157-3. 8907141

[ejp70369-bib-0083] Mendoza, M. E. , P. Sakulsriprasert , and M. P. Jensen . 2022. “Age Progression in Hypnosis for Pain and Fatigue in Individuals With Disabilities.” American Journal of Clinical Hypnosis 65: 45–59. 10.1080/00029157.2022.2060063. 35435817

[ejp70369-bib-0084] Moher, D. , A. Liberati , J. Tetzlaff , and D. G. Altman . 2009. “Preferred Reporting Items for Systematic Reviews and Meta‐Analyses: The PRISMA Statement.” PLoS Medicine 6: e1000097. 10.1371/journal.pmed.1000097. 19621072 PMC2707599

[ejp70369-bib-0085] Monolo, D. , M. Barisone , G. Cordio , et al. 2024. “The Use of Hypnotic Communication in PICC Placement: Randomized Controlled Trial Study.” American Journal of Clinical Hypnosis 66: 249–261. 10.1080/00029157.2023.2258946. 37788329

[ejp70369-bib-0086] Montgomery, G. H. , D. H. Bovbjerg , J. B. Schnur , et al. 2007. “A Randomized Clinical Trial of a Brief Hypnosis Intervention to Control Side Effects in Breast Surgery Patients.” Journal of the National Cancer Institute 99: 1304–1312. 10.1093/jnci/djm106. 17728216

[ejp70369-bib-0087] Montgomery, G. H. , C. R. Weltz , M. Seltz , and D. H. Bovbjerg . 2002. “Brief Presurgery Hypnosis Reduces Distress and Pain in Excisional Breast Biopsy Patients.” International Journal of Clinical and Experimental Hypnosis 50: 17–32. 10.1080/00207140208410088. 11778705

[ejp70369-bib-0088] Moore, L. E. , and S. L. Wiesner . 1996. “Hypnotically‐Induced Vasodilation in the Treatment of Repetitive Strain Injuries.” American Journal of Clinical Hypnosis 39: 97–104. 10.1080/00029157.1996.10403372. 8936710

[ejp70369-bib-0089] Moreno Hernández, D. , A. Téllez , T. Sánchez‐Jáuregui , C. H. García , M. García‐Solís , and A. Valdez . 2022. “Clinical Hypnosis for Pain Reduction in Breast Cancer Mastectomy: A Randomized Clinical Trial.” International Journal of Clinical and Experimental Hypnosis 70: 4–15. 10.1080/00207144.2022.2003697. 34928192

[ejp70369-bib-0090] Moretti, A. , N. Zucchini , E. Moroni , et al. 2025. “Implementation of the Enhanced Recovery After Bariatric Surgery (ERABS) Protocol With Clinical Hypnosis: Effects on Pain and Postoperative Nausea and Vomiting (PONV) Management in Bariatric Surgery.” Surgical Endoscopy 39: 7324–7335. 10.1007/s00464-025-12110-8. 40866595

[ejp70369-bib-0091] Morley, S. , C. Eccleston , and A. Williams . 1999. “Systematic Review and Meta‐Analysis of Randomized Controlled Trials of Cognitive Behaviour Therapy and Behaviour Therapy for Chronic Pain in Adults, Excluding Headache.” Pain 80: 1–13. 10.1016/s0304-3959(98)00255-3. 10204712

[ejp70369-bib-0092] Moseley, G. L. , and D. S. Butler . 2015. “Fifteen Years of Explaining Pain: The Past, Present, and Future.” Journal of Pain 16: 807–813. 10.1016/j.jpain.2015.05.005. 26051220

[ejp70369-bib-0093] Nilsson, U. , N. Rawal , B. Enqvist , and M. Unosson . 2003. “Analgesia Following Music and Therapeutic Suggestions in the PACU in Ambulatory Surgery; a Randomized Controlled Trial.” Acta Anaesthesiologica Scandinavica 47: 278–283. 10.1034/j.1399-6576.2003.00064.x. 12648193

[ejp70369-bib-0094] Nilsson, U. , N. Rawal , L. E. Uneståhl , C. Zetterberg , and M. Unosson . 2001. “Improved Recovery After Music and Therapeutic Suggestions During General Anaesthesia: A Double‐Blind Randomised Controlled Trial.” Acta Anaesthesiologica Scandinavica 45: 812–817. 10.1034/j.1399-6576.2001.045007812.x. 11472279

[ejp70369-bib-0095] Nowak, H. , N. Zech , S. Asmussen , et al. 2020. “Effect of Therapeutic Suggestions During General Anaesthesia on Postoperative Pain and Opioid Use: Multicentre Randomised Controlled Trial.” BMJ 371: m4284. 10.1136/bmj.m4284. 33303476 PMC7726311

[ejp70369-bib-0096] Ozgunay, S. E. , M. Kasapoglu Aksoy , K. N. Deniz , et al. 2024. “Effect of Hypnosis on Pain, Anxiety, and Quality of Life in Female Patients With Fibromyalgia: Prospective, Randomized, Controlled Study.” International Journal of Clinical and Experimental Hypnosis 72: 51–63. 10.1080/00207144.2023.2277853. 38060828

[ejp70369-bib-0097] Palsson, O. , M. Turner , D. Johnson , C. Burnett , and W. Whitehead . 2002. “Hypnosis Treatment for Severe Irritable Bowel Syndrome: Investigation of Mechanism and Effects on Symptoms.” Digestive Diseases and Sciences 47: 2605–2614. 12452403 10.1023/a:1020545017390

[ejp70369-bib-0098] Paredes, A. C. , P. Costa , S. Fernandes , et al. 2019. “Effectiveness of Hypnosis for Pain Management and Promotion of Health‐Related Quality‐Of‐Life Among People With Haemophilia: A Randomised Controlled Pilot Trial.” Scientific Reports 9: 13399. 10.1038/s41598-019-49827-1. 31527700 PMC6746787

[ejp70369-bib-0099] Paredes, A. C. , P. Costa , S. Roque , et al. 2021. “Effectiveness of Hypnosis for Pain and Health‐Related Quality‐Of‐Life Among People With Hemophilia: Three‐Month Outcomes of a Randomized Controlled Pilot Trial.” Complementary Therapies in Clinical Practice 45: 101486. 10.1016/j.ctcp.2021.101486. 34601386

[ejp70369-bib-0100] Patterson, D. R. , J. J. Everett , G. L. Burns , and J. A. Marvin . 1992. “Hypnosis for the Treatment of Burn Pain.” Journal of Consulting and Clinical Psychology 60: 713–717. 10.1037//0022-006x.60.5.713. 1383302

[ejp70369-bib-0101] Patterson, D. R. , M. P. Jensen , S. A. Wiechman , and S. R. Sharar . 2010. “Virtual Reality Hypnosis for Pain Associated With Recovery From Physical Trauma.” International Journal of Clinical and Experimental Hypnosis 58: 288–300. 10.1080/00207141003760595. 20509069 PMC2913598

[ejp70369-bib-0102] Patterson, D. R. , and J. T. Ptacek . 1997. “Baseline Pain as a Moderator of Hypnotic Analgesia for Burn Injury Treatment.” Journal of Consulting and Clinical Psychology 65: 60–67. 10.1037//0022-006x.65.1.60. 9103735

[ejp70369-bib-0103] Peters, S. L. , C. K. Yao , H. Philpott , G. W. Yelland , J. G. Muir , and P. R. Gibson . 2016. “Randomised Clinical Trial: The Efficacy of Gut‐Directed Hypnotherapy Is Similar to That of the Low FODMAP Diet for the Treatment of Irritable Bowel Syndrome.” Alimentary Pharmacology & Therapeutics 44: 447–459. 10.1111/apt.13706. 27397586

[ejp70369-bib-0104] Phillips‐Moore, J. S. , N. J. Talley , and M. P. Jones . 2015. “The Mind‐Body Connection in Irritable Bowel Syndrome: A Randomised Controlled Trial of Hypnotherapy as a Treatment.” Health Psychology Open 2: 2055102914564583. 10.1177/2055102914564583. 28070348 PMC5193306

[ejp70369-bib-0105] Portel, L. , A. Perel , L. Masson , C. Roy , and S. Mebs . 2022. “Tolerance's Improvement of Flexible Bronchoscopy by Ericksonian Hypnosis: The BREATH Study.” Respiratory Medicine and Research 81: 100798. 10.1016/j.resmer.2020.100798. 35584600

[ejp70369-bib-0106] Prasad, M. 2024. “Introduction to the GRADE Tool for Rating Certainty in Evidence and Recommendations.” Clinical Epidemiology and Global Health 25: 101484. 10.1016/j.cegh.2023.101484.

[ejp70369-bib-0107] R Core Team . 2017. R: A Language and Environment for Statistical Computing. R Foundation for Statistical Computing.

[ejp70369-bib-0108] Ramondo, N. , G. E. Gignac , C. F. Pestell , and S. M. Byrne . 2021. “Clinical Hypnosis as an Adjunct to Cognitive Behavior Therapy: An Updated Meta‐Analysis.” International Journal of Clinical and Experimental Hypnosis 69: 169–202. 10.1080/00207144.2021.1877549. 33646087

[ejp70369-bib-0109] Ray, K. K. , S. R. Seshasai , S. Erqou , et al. 2010. “Statins and All‐Cause Mortality in High‐Risk Primary Prevention: A Meta‐Analysis of 11 Randomized Controlled Trials Involving 65,229 Participants.” Archives of Internal Medicine 170: 1024–1031. 10.1001/archinternmed.2010.182. 20585067

[ejp70369-bib-0110] Razak, I. , T. Y. Chung , and T. S. Ahmad . 2019. “A Comparative Study of Two Modalities in Pain Management of Patients Presenting With Chronic Brachial Neuralgia.” Journal of Alternative and Complementary Medicine 25: 861–867. 10.1089/acm.2019.0052. 31211607

[ejp70369-bib-0111] Rhodes, J. R. , C. E. Corlett , M. P. Jensen , and D. R. Patterson . 2025. “Immersive Virtual Reality to Reduce Pain and Anxiety in Individuals Undergoing Orthopedic Surgery for Acute Trauma: A Randomized Clinical Trial.” International Journal of Clinical and Experimental Hypnosis 73: 106–122. 10.1080/00207144.2024.2433270. 39661916

[ejp70369-bib-0112] Rizzo, R. R. N. , F. C. Medeiros , L. G. Pires , et al. 2018. “Hypnosis Enhances the Effects of Pain Education in Patients With Chronic Nonspecific Low Back Pain: A Randomized Controlled Trial.” Journal of Pain 19: 1103. 10.1016/j.jpain.2018.03.013. 29654980

[ejp70369-bib-0113] Roberts, L. , S. Wilson , S. Singh , A. Roalfe , and S. Greenfield . 2006. “Gut‐Directed Hypnotherapy for Irritable Bowel Syndrome: Piloting a Primary Care‐Based Randomised Controlled Trial.” British Journal of General Practice 56: 115–121. PMC1828217 16464325

[ejp70369-bib-0114] Rosenbloom, B. N. , P. M. Slepian , M. A. Azam , et al. 2024. “A Randomized Controlled Trial of Clinical Hypnosis as an Opioid‐Sparing Adjunct Treatment for Pain Relief in Adults Undergoing Major Oncologic Surgery.” Journal of Pain Research 17: 45–59. 10.2147/jpr.S424639. 38196969 PMC10775151

[ejp70369-bib-0115] Rosendahl, J. , C. T. Alldredge , and A. Haddenhorst . 2024. “Meta‐Analytic Evidence on the Efficacy of Hypnosis for Mental and Somatic Health Issues: A 20‐Year Perspective.” Frontiers in Psychology 14: 1330238. 10.3389/fpsyg.2023.1330238. 38268815 PMC10807512

[ejp70369-bib-0116] Rougereau, G. , M. H. Sandiford , R. Lévêque , C. Ménigaux , T. Bauer , and A. Hardy . 2023. “Management of Anxiety for Ambulatory Hallux Valgus Surgery With a Virtual Reality Hypnosis Mask: Randomized Controlled Trial.” Foot & Ankle International 44: 539–544. 10.1177/10711007231162816. 37118916

[ejp70369-bib-0117] Sánchez‐Jáuregui, T. , A. Téllez , D. Juárez‐García , C. H. García , and F. E. García . 2019. “Clinical Hypnosis and Music in Breast Biopsy:A Randomized Clinical Trial.” American Journal of Clinical Hypnosis 61: 244–257. 10.1080/00029157.2018.1489776. 30632924

[ejp70369-bib-0118] Shahriyaripoor, R. , Z. Shahhosseini , M. Pourasghar , Z. Hoseinnezhad , and J. Ganji . 2023. “The Effect of Hypnotherapy on the Pain Intensity of Endometriosis Patients Treated With Dienogest: A Pilot Double‐Blind Randomized Clinical Trial.” Journal of Nursing and Midwifery Sciences 10: e137116.

[ejp70369-bib-0119] Shakibaei, F. , A. A. Harandi , A. Gholamrezaei , R. Samoei , and P. Salehi . 2008. “Hypnotherapy in Management of Pain and Reexperiencing of Trauma in Burn Patients.” International Journal of Clinical and Experimental Hypnosis 56: 185–197. 10.1080/00207140701849536. 18307128

[ejp70369-bib-0120] Slack, D. , L. Nelson , D. Patterson , S. Burns , K. Hakimi , and L. Robinson . 2009. “The Feasibility of Hypnotic Analgesia in Ameliorating Pain and Anxiety Among Adults Undergoing Needle Electromyography.” American Journal of Physical Medicine & Rehabilitation 88: 21–29. 10.1097/PHM.0b013e31818e00bd. 18971768 PMC5661975

[ejp70369-bib-0121] Spanos, N. P. , S. J. Liddy , H. Scott , et al. 1993. “Hypnotic Suggestion and Placebo for the Treatment of Chronic Headache in a University Volunteer Sample.” Cognitive Therapy and Research 17: 191–205. 10.1007/BF01172965.

[ejp70369-bib-0122] Spinhoven, P. , A. C. Linssen , R. Van Dyck , and F. G. Zitman . 1992. “Autogenic Training and Self‐ Hypnosis in the Control of Tension Headache.” General Hospital Psychiatry 14: 408–415. 10.1016/0163-8343(92)90008-x. 1473711

[ejp70369-bib-0123] Stam, H. J. , P. A. McGrath , R. I. Brooke , and F. Cosier . 1986. “Hypnotizability and the Treatment of Chronic Facial Pain.” International Journal of Clinical and Experimental Hypnosis 34: 182–191. 10.1080/00207148608406984. 3744611

[ejp70369-bib-0124] Starosta, A. J. , C. H. Bombardier , F. Kahlia , et al. 2024. “Feasibility of Brief, Hypnotic Enhanced Cognitive Therapy for SCI‐Related Pain During Inpatient Rehabilitation.” Archives of Physical Medicine and Rehabilitation 105: 1–9. 10.1016/j.apmr.2023.06.005. 37364685

[ejp70369-bib-0125] Stegenga, J. 2018. “Medical Nihilism.” In Handbook of the Philosophy of Medicine, edited by T. Schramme and M. Walker , 1–12. Springer Netherlands. 10.1007/978-94-017-8706-2_96-1.

[ejp70369-bib-0126] Sterne, J. A. C. , J. Savović , M. J. Page , et al. 2019. “RoB 2: A Revised Tool for Assessing Risk of Bias in Randomised Trials.” BMJ 366: l4898. 10.1136/bmj.l4898. 31462531

[ejp70369-bib-0127] Sterne, J. A. C. , A. J. Sutton , J. P. A. Ioannidis , et al. 2011. “Recommendations for Examining and Interpreting Funnel Plot Asymmetry in Meta‐Analyses of Randomised Controlled Trials.” BMJ 343: d4002. 10.1136/bmj.d4002. 21784880

[ejp70369-bib-0128] Strijkers, R. H. W. , M. Schreijenberg , H. Gerger , B. W. Koes , and A. Chiarotto . 2021. “Effectiveness of Placebo Interventions for Patients With Nonspecific Low Back Pain: A Systematic Review and Meta‐Analysis.” Pain 162: 2792–2804. 10.1097/j.pain.0000000000002272. 33769366

[ejp70369-bib-0129] Sullivan, M. D. , and J. C. Ballantyne . 2023. “The Right to Pain Relief and Other Deep Roots of the Opioid Epidemic.” Historische Zeitschrift 322: 518–520. 10.1515/hzhz-2026-1113.

[ejp70369-bib-0130] Syrjala, K. L. , C. Cummings , and G. W. Donaldson . 1992. “Hypnosis or Cognitive Behavioral Training for the Reduction of Pain and Nausea During Cancer Treatment: A Controlled Clinical Trial.” Pain 48: 137–146. 10.1016/0304-3959(92)90049-h. 1350338

[ejp70369-bib-0131] Tastan, K. , O. Ozer Disci , and T. Set . 2018. “A Comparison of the Efficacy of Acupuncture and Hypnotherapy in Patients With Migraine.” International Journal of Clinical and Experimental Hypnosis 66: 371–385. 10.1080/00207144.2018.1494444. 30152732

[ejp70369-bib-0132] Téllez, A. , T. Sánchez‐Jáuregui , D. M. Juárez‐García , and M. García‐Solís . 2016. “Breast Biopsy: The Effects of Hypnosis and Music.” International Journal of Clinical and Experimental Hypnosis 64: 456–469. 10.1080/00207144.2016.1209034. 27585728

[ejp70369-bib-0133] Ter Kuile, M. M. , P. Spinhoven , C. G. A. Linssen , F. G. Zitman , R. Van Dyck , and H. G. M. Rooijmans . 1994. “Autogenic Training and Cognitive Self‐Hypnosis for the Treatment of Recurrent Headaches in Three Different Subject Groups.” Pain 58: 331–340. 10.1016/0304-3959(94)90127-9. 7838582

[ejp70369-bib-0134] Tezcan, B. , D. Tatlısuluoglu , M. Can , et al. 2020. “A Randomized Clinical Trial on the Effect of Hypnosis on Anxiety and Pain in Rigid Cystoscopy Patients.” Journal of Endourology 35: 47–53. 10.1089/end.2020.0101. 32867544

[ejp70369-bib-0135] Thompson, T. , D. B. Terhune , C. Oram , et al. 2019. “The Effectiveness of Hypnosis for Pain Relief: A Systematic Review and Meta‐Analysis of 85 Controlled Experimental Trials.” Neuroscience and Biobehavioral Reviews 99: 298–310. 10.1016/j.neubiorev.2019.02.013. 30790634

[ejp70369-bib-0136] Tonye‐Geoffroy, L. , S. Mauboussin Carlos , S. Tuffet , et al. 2021. “Efficacy of a Combination of Hypnosis and Transcutaneous Electrical Nerve Stimulation for Chronic Non‐Cancer Pain: A Randomized Controlled Trial.” Journal of Advanced Nursing 77: 2875–2886. 10.1111/jan.14833. 33783846 PMC8251595

[ejp70369-bib-0137] Torem, M. S. 1992. ““Back From the Future”: A Powerful Age‐Progression Technique.” American Journal of Clinical Hypnosis 35: 81–88. 10.1080/00029157.1992.10402990. 1442647

[ejp70369-bib-0138] Turk, D. C. 2003. “Cognitive‐Behavioral Approach to the Treatment of Chronic Pain Patients.” Regional Anesthesia and Pain Medicine 28: 573–579. 10.1016/s1098-7339(03)00392-4. 14634950

[ejp70369-bib-0139] U.S. Burden of Disease Collaborators . 2013. “The State of US Health, 1990‐2010: Burden of Diseases, Injuries, and Risk Factors.” JAMA 310: 591–608. 10.1001/jama.2013.13805. 23842577 PMC5436627

[ejp70369-bib-0140] VanDyck, R. , F. G. Zitman , A. C. Linssen , and P. Spinhoven . 1991. “Autogenic Training and Future Oriented Hypnotic Imagery in the Treatment of Tension Headache: Outcome and Process.” International Journal of Clinical and Experimental Hypnosis 39: 6–23. 10.1080/00207149108409615. 1705921

[ejp70369-bib-0141] Vanhaudenhuyse, A. , A. Gillet , N. Malaise , et al. 2015. “Efficacy and Cost‐Effectiveness: A Study of Different Treatment Approaches in a Tertiary Pain Centre.” European Journal of Pain 19: 1437–1446. 10.1002/ejp.674. 25711348

[ejp70369-bib-0142] Veehof, M. M. , H. R. Trompetter , E. T. Bohlmeijer , and K. M. Schreurs . 2016. “Acceptance‐ and Mindfulness‐Based Interventions for the Treatment of Chronic Pain: A Meta‐Analytic Review.” Cognitive Behaviour Therapy 45: 5–31. 10.1080/16506073.2015.1098724. 26818413

[ejp70369-bib-0143] Venkiteswaran, A. , and S. Tandon . 2021. “Role of Hypnosis in Dental Treatment: A Narrative Review.” Journal of International Society of Preventive and Community Dentistry 11: 115–124. 10.4103/jispcd.JISPCD_320_20. 34036071 PMC8118047

[ejp70369-bib-0144] Viechtbauer, W. 2005. “Bias and Efficiency of Meta‐Analytic Variance Estimators in the Random‐ Effects Model.” Journal of Educational and Behavioral Statistics 30: 261–293. 10.3102/10769986030003261.

[ejp70369-bib-0145] Viechtbauer, W. 2010. “Conducting Meta‐Analyses in R With the Metafor Package.” Journal of Statistical Software 36: 1–48. 10.18637/jss.v036.i03.

[ejp70369-bib-0146] Vlaeyen, J. W. S. , and S. J. Linton . 2000. “Fear‐Avoidance and Its Consequences in Chronic Musculoskeletal Pain: A State of the Art.” Pain 85: 317–332. 10.1016/s0304-3959(99)00242-0. 10781906

[ejp70369-bib-0147] Waisblat, V. , B. Langholz , F. Bernard , et al. 2017. “Impact of a Hypnotically‐Based Intervention on Pain and Fear in Women Undergoing Labor.” International Journal of Clinical and Experimental Hypnosis 65: 64–85. 10.1080/00207144.2017.1246876. 27935457

[ejp70369-bib-0148] Werner, A. , C. Wu , R. Zachariae , E. A. Nohr , N. Uldbjerg , and M. Hansen Å . 2020. “Effects of Antenatal Hypnosis on Maternal Salivary Cortisol During Childbirth and Six Weeks Postpartum‐A Randomized Controlled Trial.” PLoS One 15: e0230704. 10.1371/journal.pone.0230704. 32357152 PMC7194394

[ejp70369-bib-0149] Williams, A. C. C. , E. Fisher , L. Hearn , and C. Eccleston . 2020. “Psychological Therapies for the Management of Chronic Pain (Excluding Headache) in Adults.” Cochrane Database of Systematic Reviews 8: Cd007407. 10.1002/14651858.CD007407.pub4. 32794606 PMC7437545

[ejp70369-bib-0150] Williams, R. M. , M. A. Day , D. M. Ehde , et al. 2022. “Effects of Hypnosis vs Mindfulness Meditation vs Education on Chronic Pain Intensity and Secondary Outcomes in Veterans: A Randomized Clinical Trial.” Pain 163: 1905–1918. 10.1097/j.pain.0000000000002586. 35082248 PMC11089905

[ejp70369-bib-0151] Wright, B. R. , and P. D. Drummond . 2000. “Rapid Induction Analgesia for the Alleviation of Procedural Pain During Burn Care.” Burns 26: 275–282. 10.1016/s0305-4179(99)00134-5. 10741595

[ejp70369-bib-0152] Yerzhan, A. , A. Ayazbekova , D. R. Lavage , and J. E. Chelly . 2025. “The Use of Medical Hypnosis to Prevent and Treat Acute and Chronic Pain: A Systematic Review and Meta‐Analysis.” Journal of Clinical Medicine 14: 4661. 10.3390/jcm14134661. 40649035 PMC12250368

[ejp70369-bib-0153] Zitman, F. G. , R. van Dyck , P. Spinhoven , and A. C. Linssen . 1992. “Hypnosis and Autogenic Training in the Treatment of Tension Headaches: A Two‐Phase Constructive Design Study With Follow‐Up.” Journal of Psychosomatic Research 36: 219–228. 10.1016/0022-3999(92)90086-h. 1564674

