# Supplementary material for: A Systematic Review of Hypnosis for Clinical Pain Relief
Source: Eur J Pain. 2026 Sep 2;30(8):e70369. doi: 10.1002/ejp.70369 (PMC13535766; doi:10.1002/ejp.70369)
Supplement: Supplementary file 1 [file EJP-30-0-s001.docx]

**Supplementary Material 2.** Summary of Risk-of-Bias (RoB) Assessment for all 21 criteria

| **No.** | **Description of Criteria** | **Cohen’s Kappa** | **Interrater agreement^#^** |
| --- | --- | --- | --- |
| 1 | Rationale/ Objective reported | 1.00* | Perfect |
| 2 | Participant source reported | 0.67* | Moderate |
| 3 | Recruitment method reported | 0.40* | Moderate |
| 4 | Inclusion/ Exclusion criteria reported | 0.65* | Moderate |
| 5 | Inclusion/ Exclusion criteria met | 0.05 | None |
| 6 | Pre-existing Pain reported | 0.09* | None |
| 7 | Abstained from medication or reported analgesic use | 0.39* | Minimal |
| 8 | Pain assessment method reported | ~0.00* | Almost perfect  (98.1% agreement) |
| 9 | Hypnotic intervention protocol | 0.20* | None |
| 10 | Control condition protocol | ~0.00* | Almost perfect  (98.1% agreement) |
| 11 | Hypnotisability measured | 0.85* | Strong |
| 12 | Matching | 0.08* | None |
| 13 | Participant characteristics reported | -0.02 | Disagreement |
| 14 | Complete Outcome data reported | 0.08 | None |
| 15 | Random allocation | 0.21* | Minimal |
| 16 | Adequate Training for hypnotists/ therapists | 0.22* | Minimal |
| 17 | Active engagement measured | 0.08* | None |
| 18 | Deviations from protocol reported | 0.39* | Minimal |
| 19 | Blind investigator | 0.47* | Weak |
| 20 | Blind data collection | 0.62* | Moderate |
| 21 | Treatment expectation measured | 0.60* | Moderate |

* *p-value* < .05

**^#^** Cohen’s Kappa thresholds

Less than 0: Disagreement

0.00–0.20: No agreement

0.21–0.39: Minimal agreement

0.40–0.59: Weak agreement

0.60–0.79: Moderate agreement

0.80–0.89: Strong agreement

0.81–1.00: Perfect/Almost perfect agreement
