# Supplementary material for: A Systematic Review of Hypnosis for Clinical Pain Relief
Source: Eur J Pain. 2026 Sep 2;30(8):e70369. doi: 10.1002/ejp.70369 (PMC13535766; doi:10.1002/ejp.70369)
Supplement: Supplementary file 3 [file EJP-30-0-s003.docx]

**Supplementary Material 4.** GRADE Level of Evidence Assessment

|  | **Initial rating** | **Study design and execution** | **Inconsistency** | **Indirectness** | **Imprecision** | **Dissemination bias** |
| --- | --- | --- | --- | --- | --- | --- |
| **Rating Process** | High certainty | No downgrade | Downgrade for some | No downgrade | Downgrade for some | Not detected |
| **Explanation** | *Initial rating*: Participants were randomly assigned to experimental and control interventions. Therefore, the body of evidence received a high initial rating.  *Study design and execution*: In the process of assessing risk of bias, more than half the studies did not report treatment expectations (92 studies, 86.8%), active engagement in hypnosis (85 studies, 80.2%), hypnotisability (84 studies, 79.2%), blinding of investigator (71 studies, 67.0%) or data collection (65 studies, 61.3%) or abstinence from medication (56 studies, 52.8%). However, sensitivity analysis by removing high risk-of-bias studies did not lead to changes in findings. Therefore, the level of evidence was not downgraded.  *Inconsistency*: We consider the conclusions of most meta-analyses unclear on the basis of inconsistency as most meta-analyses except for one (Hypnosis versus CBT on post-intervention pain intensity: moderate heterogeneity) had substantial to high between-study heterogeneity. Therefore, the level of evidence was downgraded.  *Indirectness*: All studies looked at adult populations experiencing clinical pain, utilised hypnosis as a primary intervention, included a control intervention and measured pain intensity as an outcome. Therefore, the methodology of our included studies adequately addressed the research question and the level of evidence was not downgraded.  *Imprecision*: Only one meta-analysis had a large sample size (hypnosis versus non-active control on post-intervention pain: 4,572 participants) and statistically significant standardised mean differences. However, we considered most included studies (78 studies, 73.58%) to have limited sample sizes (i.e., less than 100 participants). In order to detect subtle analgesic effects of hypnosis or control interventions, especially when baseline pain intensity is low, we presume large sample sizes to be required. Therefore, the level of evidence was downgraded.  *Dissemination Bias*: Visual inspection of funnel plots, where possible (i.e., sample size of 10 or more), did not reveal publication biases. Egger’s regression test of our largest meta-analysis (hypnosis versus non-active control on post-intervention pain) did not reveal small study effects. Therefore, the level of evidence was not downgraded.  *Magnitude of Effect Size*: Effect sizes were mostly small differences. Therefore, we did not upgrade the level of evidence.  *Dose‐response Gradient*: Not applicable  *Opposite Residual Confounding*: For comparisons involving active control interventions (i.e., relaxation, pain education, and CBT), the control interventions are themselves known to reduce pain and, in some cases, include elements that overlap with hypnotic procedures. This means any unmeasured confounding would likely bias the effect estimate toward the null rather than exaggerate benefit. Therefore, we did not upgrade the level of evidence.  *Final rating*: Low for most. However, the primary meta-analysis comparing the effect of hypnosis and non-active controls on post-intervention pain, is likely to have a moderate-high level of evidence due to high precision, albeit moderate inconsistency. | | | | | |
