# Supplementary material for: A Systematic Review of Hypnosis for Clinical Pain Relief
Source: Eur J Pain. 2026 Sep 2;30(8):e70369. doi: 10.1002/ejp.70369 (PMC13535766; doi:10.1002/ejp.70369)
Supplement: Supplementary file 4 [file EJP-30-0-s004.docx]

**Supplementary Material 5.** Additional Materials.

**Databases and search strategy**

Two consecutive searches (an initial search and an updated search) were conducted across five databases from inception to 20 October 2025: PsycINFO, PubMed, Scopus, Web of Science, and Cochrane Library Registrar of Controlled Trials. Search terms included variants of ‘hypnotic intervention’ (“hypnosis” or “hypnotherapy” or “hypnoses” or “hypnotism” or “hypnotherapies” or “hypnotic” or “suggestion”) or ‘non-pharmacological intervention’ (“non-pharmacological treatment” or “non-pharmacological analgesia” or “non-pharmacological anaesthesia” or “non-pharmacological anesthesia” or “non-pharmacological pain relief”) and ‘pain reduction’ (“pain relief” or “pain control” or “pain management” or “analgesi*”) to maximise retrieval of relevant articles. The search terms were applied to the title, keywords, and abstract for all databases except for Scopus, where the search was restricted to the title only to improve search specificity.

**Hypnosis modality coding**

The modality of hypnosis adopted was coded into five categories: in-person therapist-delivered hypnosis only, in-person audiotaped hypnosis only, self-hypnosis training only, in-person therapist-delivered hypnosis with self-practice/self-hypnosis between sessions and virtual-reality-delivered hypnosis.

**Narrative synthesis: Hypnosis versus music**

Seven studies examined the effect of hypnosis on pain relief vis-à-vis a music or music with hypnosis control group. Two studies comprising 157 participants reported no significant difference in pain intensity reduction when comparing hypnosis with listening to music only (Sánchez-Jáuregui et al., 2019; Bicego et al., 2021). In contrast, two studies comprising 200 participants reported that hypnosis produced significantly lower post-operative pain intensity when compared to music alone (Mackey, 2010; Mackey, 2018). Three studies comprising 224 participants reported no significant differences in pain intensity reduction when comparing listening to music with hypnosis suggestions versus listening to music alone (Nilsson et al., 2001; Nilsson et al., 2003; Téllez et al., 2016).

**Narrative synthesis: Hypnosis versus mindfulness**

Four studies comprising 610 participants reported no statistically significant difference between hypnosis and mindfulness when it came to reducing pain intensity (Garland et al., 2017; Hanley et al., 2021; Williams et al., 2022; Anastas et al., 2024). In a pilot trial, mindfulness training combined with hypnosis was associated with reductions in pain intensity with a small effect size difference (*d*=-0.36); these improvements were modest and not clearly greater than those observed with hypnosis alone (*d*=-0.43) (Jensen et al., 2018a).

**Narrative synthesis: Hypnosis versus other psychological interventions**

Hypnotic intervention was reported as not providing significantly greater pain relief than stress-reduction training (Frenay et al., 2001). Hypnosis with self-care was also reported to be no more effective in reducing chronic pain intensity compared to self-care alone, both immediately post-intervention and at the six-month follow-up (Bicego et al., 2021).

**Narrative synthesis: Hypnosis versus physiological treatments**

Due to a small number of studies, findings on hypnosis effect on reducing pain as compared to other physiological treatments were inconclusive and reported mixed results. Two studies reported a non-significant lower post-intervention chronic pain intensity following self-hypnosis compared to massage physiotherapy (Vanhaudenhuyse et al., 2015), while in contrast, another study reported that older adults who received hypnosis experienced a greater decrease in average pain compared to massage (Ardigo et al., 2016). Comparing hypnosis and acupuncture, one study reported no significant differences between the post-intervention pain intensity among patients with migraine (Tastan et al., 2018), while another study found hypnosis to be more effective (larger effect size) in reducing chronic brachial neuralgia pain intensity, although both interventions delivered large effect sizes after 16 weeks of intervention (hypnosis: *d*=-1.60; acupressure: *d*=-1.01) (Razak et al., 2019). One study reported a non-significant reduction in non-cancer pain intensity following practitioner-delivered hypnosis with TENS as compared to TENS alone (Tonye-Geoffroy et al., 2021), and another study also found that the addition of biofeedback to hypnosis led to a greater analgesic effect (larger effect size; *d*=-1.01) as compared to hypnosis alone (*d*=-0.43) (Jensen et al., 2018b). Lastly, specifically for irritable bowel syndrome, hypnosis was not significantly different from a low FODMAP diet in reducing abdominal pain intensity at a three- and six-months follow-up (Peters et al., 2016).

**References**

Anastas TM, Turner AP, Ho EJ, Day MA, Ehde DM, Jensen MP, Williams RM. Evaluating the benefits of a second pain treatment following a clinical trial. Rehabil Psychol 2024;69:74-83.

Ardigo S, Herrmann FR, Moret V, Déramé L, Giannelli S, Gold G, Pautex S. Hypnosis can reduce pain in hospitalized older patients: a randomized controlled study. BMC Geriatr 2016;16:14.

Bicego A, Monseur J, Collinet A, Donneau AF, Fontaine R, Libbrecht D, Malaise N, Nyssen AS, Raaf M, Rousseaux F, Salamun I, Staquet C, Teuwis S, Tomasella M, Faymonville ME, Vanhaudenhuyse A. Complementary treatment comparison for chronic pain management: a randomized longitudinal study. PLoS One 2021;16:e0256001.

Frenay MC, Faymonville ME, Devlieger S, Albert A, Vanderkelen A. Psychological approaches during dressing changes of burned patients: a prospective randomised study comparing hypnosis against stress reducing strategy. Burns 2001;27:793-9.

Garland EL, Baker AK, Larsen P, Riquino MR, Priddy SE, Thomas E, Hanley AW, Galbraith P, Wanner N, Nakamura Y. Brief mindfulness training and hypnotic suggestion for acute pain relief: a randomized controlled trial. J Gen Intern Med 2017;32:1106-13.

Hanley AW, Gililland J, Erickson J, Pelt C, Peters C, Rojas J, Garland EL. Brief preoperative mind-body therapies for total joint arthroplasty patients: a randomized controlled trial. Pain 2021;162:1749-57.

Jensen MP, Battalio SL, Chan JF, Edwards KA, Day MA, Sherlin LH, Ehde DM. Use of neurofeedback and mindfulness to enhance response to hypnosis treatment in individuals with multiple sclerosis: results from a pilot randomized clinical trial. Int J Clin Exp Hypn 2018a;66:231-64.

Jensen MP, Battalio SL, Chan JF, Edwards KA, Day MA, Sherlin LH, Ehde DM. Use of neurofeedback and mindfulness to enhance response to hypnosis treatment in individuals with multiple sclerosis: results from a pilot randomized clinical trial. Int J Clin Exp Hypn 2018b;66:231-64.

Mackey EF. Effects of hypnosis as an adjunct to intravenous sedation for third molar extraction: a randomized, blind, controlled study. Int J Clin Exp Hypn 2010;58:21-38.

Mackey EF. An extension study using hypnotic suggestion as an adjunct to intravenous sedation. Am J Clin Hypn 2018;60:378-85.

Nilsson U, Rawal N, Uneståhl LE, Zetterberg C, Unosson M. Improved recovery after music and therapeutic suggestions during general anaesthesia: a double-blind randomised controlled trial. Acta Anaesthesiol Scand 2001;45:812-17.

Nilsson U, Rawal N, Enqvist B, Unosson M. Analgesia following music and therapeutic suggestions in the PACU in ambulatory surgery; a randomized controlled trial. Acta Anaesthesiol Scand 2003;47:278-83.

Peters SL, Yao CK, Philpott H, Yelland GW, Muir JG, Gibson PR. Randomised clinical trial: the efficacy of gut-directed hypnotherapy is similar to that of the low FODMAP diet for the treatment of irritable bowel syndrome. Aliment Pharmacol Ther 2016;44:447-59.

Razak I, Chung TY, Ahmad TS. A comparative study of two modalities in pain management of patients presenting with chronic brachial neuralgia. J Altern Complement Med 2019;25:861-7.

Sánchez-Jáuregui T, Téllez A, Juárez-García D, García CH, García FE. Clinical hypnosis and music in breast biopsy: a randomized clinical trial. Am J Clin Hypn 2019;61:244-57.

Tastan K, Ozer Disci O, Set T. A comparison of the efficacy of acupuncture and hypnotherapy in patients with migraine. Int J Clin Exp Hypn 2018;66:371-85.

Téllez A, Sánchez-Jáuregui T, Juárez-García DM, García-Solís M. Breast biopsy: the effects of hypnosis and music. Int J Clin Exp Hypn 2016;64(4):456-69.

Tonye-Geoffroy L, Mauboussin Carlos S, Tuffet S, Fromentin H, Berard L, Leblanc J, Laroche F. Efficacy of a combination of hypnosis and transcutaneous electrical nerve stimulation for chronic non-cancer pain: a randomized controlled trial. J Adv Nurs 2021;77(6):2875-86.

Vanhaudenhuyse A, Gillet A, Malaise N, Salamun I, Barsics C, Grosdent S, Maquet D, Nyssen AS, Faymonville ME. Efficacy and cost-effectiveness: a study of different treatment approaches in a tertiary pain centre. Eur J Pain 2015;19:1437-46.

Williams RM, Day MA, Ehde DM, Turner AP, Ciol MA, Gertz KJ, Patterson D, Hakimian S, Suri P, Jensen MP. Effects of hypnosis vs mindfulness meditation vs education on chronic pain intensity and secondary outcomes in veterans: a randomized clinical trial. Pain 2022;163:1905-18.
