# Supplementary material for: A Systematic Review of Hypnosis for Clinical Pain Relief
Source: Eur J Pain. 2026 Sep 2;30(8):e70369. doi: 10.1002/ejp.70369 (PMC13535766; doi:10.1002/ejp.70369)
Supplement: Supplementary file 5 [file EJP-30-0-s005.docx]

**Supplementary Material 3. Summary of Risk-of-Bias (RoB) Assessment for all 106 included studies**

| **No.** | **Author/Year** | **Criteria met (out of 21)** | **Final RoB** |
| --- | --- | --- | --- |
| 1 | Stam et al., 1986 | 10 | High |
| 2 | Evans & Richardson, 1988 | 13 | Moderate |
| 3 | VanDyck et al., 1991 | 14 | Moderate |
| 4 | Liu et al., 1992 | 15 | Low |
| 5 | Patterson et al., 1992 | 18 | Low |
| 6 | Spinhoven et al., 1992 | 17 | Low |
| 7 | Syrjala et al., 1992 | 11 | Moderate |
| 8 | Zitman et al., 1992 | 15 | Low |
| 9 | Everett et al., 1993 | 13 | Moderate |
| 10 | Spanos et al., 1993 | 18 | Low |
| 11 | Ter Kuile et al., 1994 | 18 | Low |
| 12 | Melzack et al., 1996 | 15 | Low |
| 13 | Moore & Wiesner, 1996 | 14 | Moderate |
| 14 | Patterson & Ptacek, 1997 | 15 | Low |
| 15 | Ghoneim et al., 2000 | 12 | Moderate |
| 16 | Wright & Drummond, 2000 | 11 | Moderate |
| 17 | Frenay et al., 2001 | 13 | Moderate |
| 18 | Nilsson et al., 2001 | 15 | Low |
| 19 | Gay et al., 2002 | 17 | Low |
| 20 | Montgomery et al., 2002 | 15 | Low |
| 21 | Palsson et al., 2002 | 13 | Moderate |
| 22 | Ginandes et al., 2003 | 17 | Low |
| 23 | Nilsson et al., 2003 | 13 | Moderate |
| 24 | Jones et al., 2006 | 14 | Moderate |
| 25 | Roberts et al., 2006 | 15 | Low |
| 26 | Askay et al., 2007 | 14 | Moderate |
| 27 | Castel et al., 2007 | 15 | Low |
| 28 | Montgomery et al., 2007 | 16 | Low |
| 29 | Abrahamsen et al., 2008 | 16 | Low |
| 30 | Marc et al., 2008 | 16 | Low |
| 31 | Shakibaei et al., 2008 | 13 | Moderate |
| 32 | Abrahamsen et al., 2009 | 15 | Low |
| 33 | Castel et al., 2009 | 12 | Moderate |
| 34 | Jensen et al., 2009A | 15 | Low |
| 35 | Jensen et al., 2009B | 15 | Low |
| 36 | Slack et al., 2009 | 12 | Moderate |
| 37 | Mackey, 2010 | 13 | Moderate |
| 38 | Patterson et al., 2010 | 12 | Moderate |
| 39 | Castel et al., 2012 | 13 | Moderate |
| 40 | Lindfors et al., 2012 | 16 | Low |
| 41 | Guittier et al., 2013 | 14 | Moderate |
| 42 | Phillips-Moore et al., 2015 | 18 | Low |
| 43 | Vanhaudenhuyse et al., 2015 | 12 | Moderate |
| 44 | Akgul et al., 2016 | 15 | Low |
| 45 | Ardigo et al., 2016 | 17 | Low |
| 46 | Peters et al., 2016 | 16 | Low |
| 47 | Téllez et al., 2016 | 12 | Moderate |
| 48 | Beevi et al., 2017 | 15 | Low |
| 49 | Garland et al., 2017 | 14 | Moderate |
| 50 | Hosseinzadegan et al., 2017 | 16 | Low |
| 51 | Waisblat et al., 2017 | 12 | Moderate |
| 52 | Amraoui et al., 2018 | 12 | Moderate |
| 53 | Jafarizadeh et al., 2018 | 16 | Low |
| 54 | Jensen et al., 2018 | 17 | Low |
| 55 | Mackey, 2018 | 16 | Low |
| 56 | Rizzo et al., 2018 | 16 | Low |
| 57 | Tastan et al., 2018 | 13 | Moderate |
| 58 | Lee et al., 2019 | 15 | Low |
| 59 | Paredes et al., 2019 | 17 | Low |
| 60 | Razak et al., 2019 | 10 | High |
| 61 | Sánchez-Jáuregui et al., 2019 | 12 | Moderate |
| 62 | Aravena et al., 2020 | 13 | Moderate |
| 63 | Fusco et al., 2020 | 13 | Moderate |
| 64 | Garcia et al., 2020 | 15 | Low |
| 65 | Jensen et al., 2020 | 20 | Low |
| 66 | Nowak et al., 2020 | 17 | Low |
| 67 | Werner et al., 2020 | 12 | Moderate |
| 68 | Bicego et al., 2021 | 12 | Moderate |
| 69 | Eaton et al., 2021 | 16 | Low |
| 70 | Hanley et al., 2021 | 16 | Low |
| 71 | Joo et al., 2021 | 14 | Moderate |
| 72 | Lang et al., 2021 | 15 | Low |
| 73 | Paredes et al., 2021 | 16 | Low |
| 74 | Tezcan et al., 2021 | 13 | Moderate |
| 75 | Tonye‐Geoffroy et al., 2021 | 14 | Moderate |
| 76 | Askay et al., 2022 | 16 | Low |
| 77 | Brooks et al., 2022 | 13 | Moderate |
| 78 | Coulibaly et al., 2022 | 12 | Moderate |
| 79 | Lemoine et al., 2022 | 13 | Moderate |
| 80 | Mendoza et al., 2022 | 10 | High |
| 81 | Moreno Hernández et al., 2022 | 13 | Moderate |
| 82 | Portel et al., 2022 | 15 | Low |
| 83 | Williams et al., 2022 | 19 | Low |
| 84 | Eaton et al., 2023 | 16 | Low |
| 85 | Gullo et al., 2023 | 11 | Moderate |
| 86 | Jensen et al., 2023 | 15 | Low |
| 87 | Kaczmarska et al., 2023 | 16 | Low |
| 88 | Khazraee et al., 2023 | 13 | Moderate |
| 89 | Rougereau et al., 2023 | 14 | Moderate |
| 90 | Shahriyaripoor et al., 2023 | 16 | Low |
| 91 | Anastas et al., 2024 | 17 | Low |
| 92 | Anderson et al., 2024 | 15 | Low |
| 93 | Azam et al., 2024 | 16 | Low |
| 94 | Barat et al., 2024 | 14 | Moderate |
| 95 | Chiuve et al., 2024 | 13 | Moderate |
| 96 | Dorta et al., 2024 | 16 | Low |
| 97 | Monolo et al., 2024 | 12 | Moderate |
| 98 | Ozgunay et al., 2024 | 15 | Low |
| 99 | Rosenbloom et al., 2024 | 15 | Low |
| 100 | Starosta et al., 2024 | 16 | Low |
| 101 | Aravena et al., 2025 | 17 | Low |
| 102 | Haidamous et al., 2025 | 18 | Low |
| 103 | Jensen et al., 2025 | 15 | Low |
| 104 | Maamar et al., 2025 | 15 | Low |
| 105 | Moretti et al., 2025 | 14 | Moderate |
| 106 | Rhodes et al., 2025 | 10 | High |
